# Species, Habitats, Society: An Evaluation of Research Supporting EU's Natura 2000 Network

**DOI:** 10.1371/journal.pone.0113648

**Published:** 2014-11-21

**Authors:** Viorel D. Popescu, Laurentiu Rozylowicz, Iulian M. Niculae, Adina L. Cucu, Tibor Hartel

**Affiliations:** 1 Earth to Ocean Research Group, Department of Biological Sciences, Simon Fraser University, Burnaby, British Columbia, Canada; 2 Centre for Environmental Research (CCMESI), University of Bucharest, Bucharest, Romania; 3 Department of Environmental Studies, Sapientia Hungarian University of Transylvania, Cluj-Napoca, Romania; University of Brasilia, Brazil

## Abstract

The Natura 2000 network is regarded as one of the conservation success stories in the global effort to protect biodiversity. However, significant challenges remain in Natura 2000 implementation, owing to its rapid expansion, and lack of a coherent vision for its future. Scientific research is critical for identifying conservation priorities, setting management goals, and reconciling biodiversity protection and society in the complex political European landscape. Thus, there is an urgent need for a comprehensive evaluation of published Natura 2000 research to highlight prevalent research themes, disciplinary approaches, and spatial entities. We conducted a systematic review of 572 scientific articles and conference proceedings focused on Natura 2000 research, published between 1996 and 2014. We grouped these articles into ‘ecological’ and ‘social and policy’ categories. Using a novel application of network analysis of article keywords, we found that Natura 2000 research forms a cohesive small-world network, owing to the emphasis on ecological research (79% of studies, with a strong focus on spatial conservation planning), and the underrepresentation of studies addressing ‘social and policy’ issues (typically focused on environmental impact assessment, multi-level governance, agri-environment policy, and ecosystem services valuation). ‘Ecological’ and ‘social and policy’ research shared only general concepts (e.g., Natura 2000, Habitats Directive) suggesting a disconnection between these disciplines. The UK and the Mediterranean basin countries dominated Natura 2000 research, and there was a weak correlation between number of studies and proportion of national territory protected. Approximately 40% of ‘social and policy’ research and 26% of ‘ecological’ studies highlighted negative implications of Natura 2000, while 21% of studies found positive social and biodiversity effects. We emphasize the need for designing inter- and transdisciplinary research in order to promote a social-ecological understanding of Natura 2000, and advance EU conservation policies.

## Introduction

The European Union (EU) Directive on the conservation of natural habitats and of wild fauna and flora (Habitats Directive) and the Directive on the conservation of wild birds (Birds Directive) are regarded as two of the strongest international legal tools for nature protection [1], [2]. One of the outcomes of their implementation was the creation of a set of sites across all the EU member states meant to safeguard biodiversity called the Natura 2000 network (hereafter N2K) [2]. Considerable effort was put into reaching agreement on the criteria for a coherent implementation of N2K, involving a series of biogeographical seminars to identify the levels of coverage of species and habitat types for meeting the obligations of the Habitats Directive [1]. N2K is nearing completion; in 2013 there were 23,814 terrestrial sites covering 860,511 km^2^ (Fig. S1), the world's largest ecological network united under a single, unified regulatory framework [3]. The expansion of N2K also contributes to achieving the goals of the Convention on Biological Diversity Aichi Biodiversity Targets, a complex set of measures meant to stop biodiversity loss, encourage sustainable use of natural resources, and contribute to human well-being (EU 2020 Biodiversity Strategy; http://ec.europa.eu/environment/nature/biodiversity/comm2006/2020.htm). However, despite successes in extending N2K [3], [4], and progress towards identifying threats to N2K and priorities for research [5], a coherent vision for the future of these sites is lacking [6], and obstacles remain in implementing and enforcing the Habitats and Birds Directives (e.g., conflicting conservation objectives [7], lack of coordination across member states on methodologies used to assess conservation priorities [8], low penetration of scientific information in N2K management plans [9]).

The successful implementation of N2K requires an understanding of the social and political realities in which N2K sites are embedded, and developing scientific research that addresses core conservation issues and informs future European conservation policies [2]. A recent study by Hochkirch and colleagues [6] highlighted several domains that the EU should address for a coherent implementation of the Habitats and Birds Directives. These are: (1) comprehensive scientific knowledge, (2) strategic conservation planning and adaptive management for each site, (3) biodiversity monitoring system for informing adaptive management, and (4) financial resources for implementing N2K and education on the ecological and social benefits of N2K. N2K sites often overlap with landscapes with long history of traditional human activities (i.e., cultural landscapes), productive agricultural lands, and other natural resources [10], [11]. In such landscapes, the persistence of a large number of species and habitats listed in the Birds and Habitats Directives [12], as well as a number of ecosystem services, such as provisioning of water, wood, and food, carbon sequestration, and biodiversity [13], strongly depends on the continuation of low-intensity (traditional) land management [14]. Traditional rural communities in these landscapes may still retain high levels of traditional ecological knowledge and skills to sustainably manage their natural resources [15], [16]. However, conflicts between opportunities for economic development of rural communities and conservation objectives are common [17], especially in developing countries (e.g., Eastern Europe [18]). The N2K is therefore a complex social-ecological network in which site-specific conservation targets can only be achieved when integrated and harmonized with local social desires [14]. Arguably, the human-dominated N2K landscape represents an ideal case for adopting core principles of conservation- and sustainability sciences in decision-making, along with social, political, and economic aspects [19], [20], [21].

The goal of our review is to evaluate existing research directions, as well as gaps in our understanding of N2K (e.g., research domains, taxonomic, methodological and spatial bias) in an effort to highlight research opportunities and shortcomings, and further inform the implementation of EU conservation policies. The specific objectives are: (i) to examine the distribution of N2K studies published since 1996 by discipline (‘ecological’ vs. ‘social and policy’), and spatial scale (‘regional’, ‘national’, ‘multinational’, ‘European’) across EU member states, (ii) to synthesize findings on the efficacy of N2K sites from ecological and social perspectives, by summarizing the outcomes of the research articles by discipline and spatial scale, and (iii) to identify potential gaps and opportunities in N2K scientific research using a novel application of network analysis [22], [23] of article keywords to describe current research themes and directions. We apply separate network analyses by discipline to identify commonalities and disparities between ‘ecology’ and ‘social and policy’ research, and evaluate the inter- and transdisciplinarity of N2K research to date.

## Methods

### Literature selection and classification

**Figure 1:**
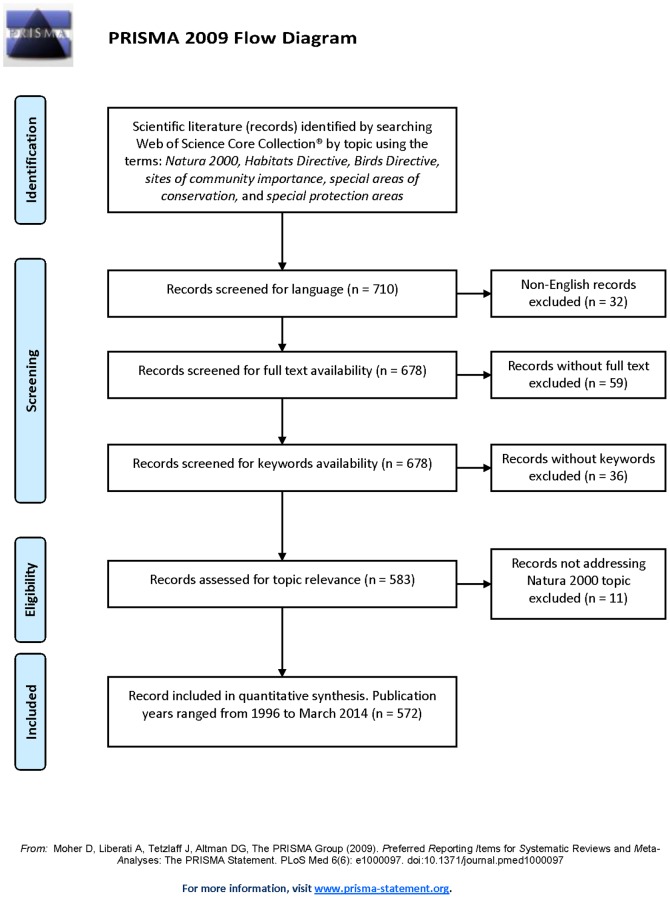
Flow diagram depicting the steps in selecting the literature reviewed in this study.

We investigated the breadth of the English-written scientific literature (peer-reviewed articles and conference proceedings) that addressed N2K between 1996 and March 2014. We searched Web of Science Core Collection (Thomson Reuters, NY) by topic (i.e., abstracts, keywords, and titles) using the following individual search terms: *Natura 2000, Habitats Directive, Birds Directive, sites of Community importance, special areas of conservation*, and *special protection areas*. We excluded articles from journals (e.g., Bird Conservation International, Journal of Environmental Law, Ocean & Coastal Management) or conference proceedings that did not require keywords, articles from journals that required individual subscription (i.e., unavailable for full-text download), the topic was not N2K-focused (i.e., did not provide or discuss data N2K species or habitats, N2K sites, European conservation policies or social aspects directly related to N2K). The search yielded 678 articles and conference proceedings, of which 106 did not fit the criteria listed above, resulting in a database of 572 papers that were accessible, had keywords, and were focused on N2K (Fig. 1, Checklist S1, Data S1, Data S2, Data S3).

We then extracted the keywords for each article to be used in the network analysis and classified the articles based on three criteria as follows:

First, we classified articles based on type of study: ‘ecology’ or ‘social and policy’. A paper was included in the category ‘ecology’ if addressed broad topics related to species, habitats and landscapes including conservation planning, assessments of the status of species and habitats (e.g., [24], [25]), expanding the N2K network for reaching taxa-specific conservation targets (e.g., [26], [27]), or assessing N2K resilience of ecological systems to climate change (e.g., [28]). A paper was included in the category of ‘social and policy’ if addressed aspects related to decision-making (e.g., [29]), evaluated the regulatory framework lending to N2K expansion, such as the adequacy of implementation of the Habitats Directive (e.g., [30], [31]), addressed stakeholder involvement in N2K management (e.g., [32], [33], [34]) or survey the level of acceptance by local communities or NGOs of implementing the Habitats and Birds Directives (e.g., [35]). While we acknowledge that social and policy studies may have different foci and research methods, we aggregated these two types of studies because typically social studies also addressed policy and *vice versa*, which made the separation between these two domains of social science difficult [36]. Second, we classified articles based on spatial extent of analysis as ‘regional’ (covering regions within a country), ‘national’, ‘multinational’ (more than one country but not the entire EU), or ‘European’ (EU or Europe in the geographic sense). Finally, we classified articles based on the outcomes of implementing N2K: ‘positive’ (e.g., N2K sites effectively protect habitats and species, N2K policies are effective for protecting biodiversity, people support N2K policies), ‘negative’ (e.g., N2K is not efficient for protecting habitats or species of Community interest, the N2K fail to represent some biogeographical areas, N2K is negatively perceived by people) and ‘mixed’ (e.g., outcomes are good for some taxa, but not others), or ‘no opinion’ (or ‘neutral’, when the performance or social acceptance of N2K were not explicitly tested; e.g., new monitoring methods in N2K, guidelines for site selection [37]) (see Table S1 for examples for positive and negative outcomes). Additionally, we summarized positive and negative study findings and recommendations to highlight inconsistencies, as well as performance of N2K implementation.

**Figure 2:**
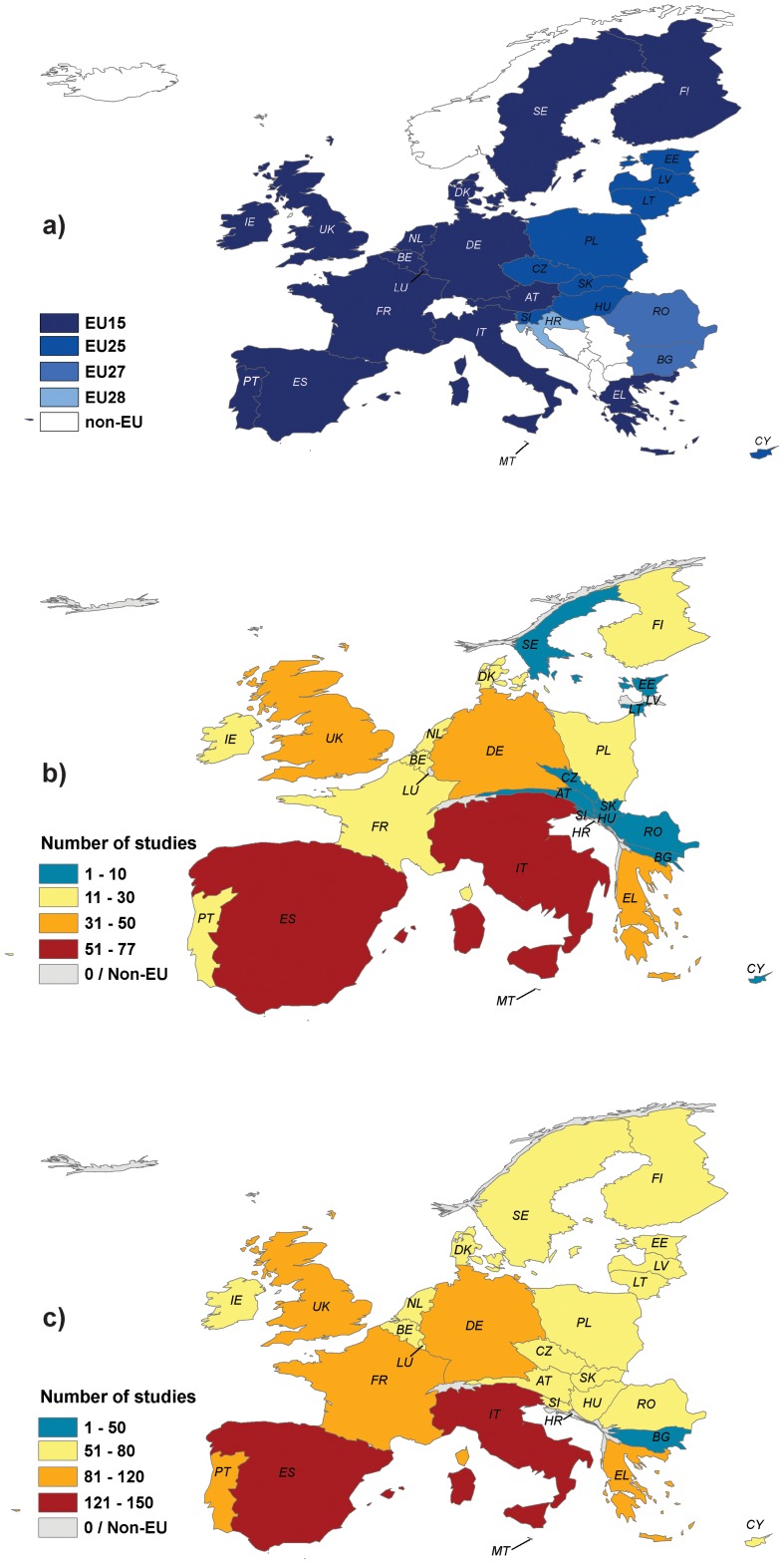
Distribution of N2K research in the European Union, 1996–2014: (a) European Union member states as of 2014; (b) Number of N2K studies with regional, national, and multinational scope; (c) total number of N2K studies (regional, national, multinational, and EU-wide scope). Country size represents relative contribution to number of N2K studies in (b) and (c) (created using the Gastner/Newman diffusion-based algorithm). (AT  =  Austria, BE  =  Belgium, BG  =  Bulgaria, CY  =  Cyprus, CZ  =  Czech Republic, DE  =  Germany, DK  =  Denmark, EE  =  Estonia, EL  =  Greece, ES  =  Spain, FI  =  Finland, FR  =  France, HR  =  Croatia, IE  =  Ireland, IT  =  Italy, LT  =  Lithuania, LU  =  Luxembourg, LV  =  Latvia, MT  =  Malta, NL  =  Netherlands, PL  =  Poland, PT  =  Portugal, RO  =  Romania, SE  =  Sweden, SI  =  Slovenia, SK  =  Slovakia, UK  =  United Kingdom).

We were also interested in understanding the spatial focus of N2K studies and highlighting potential research disparities by country and year of EU accession. We tested whether EU countries that joined the European Union earlier (EU15 states) had a greater body of N2K research compared to those that joined the EU later (EU25 states) (Fig. 2a) using Kruskal-Wallis non-parametric tests. We also examined the correlation between the number of studies and the percentage of national territory in N2K using Spearman rank correlations. While it may seem obvious that older member states would have more N2K-focused studies, the reality is more complex. Studies only started to accumulate after 2004, which coincided with the EU25 enlargement. At the same time, the number of N2K sites only started to increase substantially in EU15 after 2000, and the expansion (in terms of area protected) was not equal among member states [1]. In fact, EU25 countries lagged behind EU15 by a relatively small time period (3–5 years) in declaring N2K sites, and the inherent differences between countries in the adoption of N2K, makes the case for investigating differences in N2K research between EU15 and EU25 countries. Only two countries belonged to EU27 (Romania and Bulgaria), which did not ensure enough replication for adding EU27 as a factor in the analysis. Croatia, which became the 28^th^ EU member in July 2013, was not included in the study. We then mapped regional disparities in the number of N2K studies using ScapeToad (http://scapetoad.choros.ch/), which uses the Gastner/Newman diffusion-based algorithm [38] to adapt map surfaces to user-defined variables (here, number of studies per EU country) without altering their topological relations.

### Network analysis of keywords

Network analysis is used in the field of ecology and evolution for quantifying associations between objects such as molecules, individuals, or species [39], [40], [41], making it possible to understand interactions and emergent properties of complex systems. In our study, standardized keywords (see File S1) are nodes in the network, and are paired using an undirected link if they appear in the same paper. If the same pair of keywords is repeated in another article, the weight of the link is incremented with one step. This process is then repeated across all keywords, resulting in an undirected, weighted network [23]. We built three keyword networks: one across all studies and one for each article focus (i.e., ‘ecological’ and ‘social and policy’).

We analyzed the keywords in the network using two measures of centrality: (1) degree, which measures the number of relations that run from a keyword to other keywords, and (2) betweenness, which measures the number of shortest paths that run through a keyword, thus identifying keywords that connect other keywords that would otherwise be disconnected or distantly connected. High betweenness keywords might have a lesser degree, but they act as hubs for connecting different articles or areas of study (see File S1 for full description of metrics used in this study). Along with keyword-specific measures, we analyzed the network structure using network-specific measures: (1) density, which is the proportion of keyword pairs out of the total number of possible pairs in the network, and (2) clustering coefficient, which is a synthetic measure of the degree to which keywords tend to aggregate in the network.

We built the networks using NodeXL and Gephi software for network analysis and visualization [42], [43]. We first evaluated the characteristics of keyword networks using the node- and network-level measures. We identified important keywords based on their degree and keywords acting as connectors between articles/fields based on their betweenness. We then contrasted the three networks with random networks built with similar number of keywords and similar average number of edges per vertex. Small-world phenomena such as social networks and functional scientific communities [44] are characterized by a clustering coefficient significantly higher than expected under complete randomness and a similar average path length (distance) [45]. Despite the relatively recent advent of N2K studies, we expected that the overall N2K keyword network would behave like a “small-world” network, which includes a relatively small number of recurring keywords denoting important study topics or areas that connect a majority of low-frequency keywords.

## Results

### Study foci and spatial coverage

We reviewed a total of 572 articles and conference proceedings published between 1996 and March 2014 (Data S1, Data S2, Data S3). N2K studies have only started to accumulate since 2004; between 1996 and 2003 there were only 1–9 N2K studies published per year. 452 studies were categorized as ‘ecological’ (79%), and 120 studies as ‘policy and social’ (21%).

Of the total, 320 studies focused on ‘regional’ scale (55.9%), while 129 were ‘national’ (22.5%), 70 were ‘multinational’ (12.2%), and 53 were ‘European’ (9.2%). Overall, the highest number of regional, national, and multinational studies addressed N2K in Italy (77), Spain (66), the UK (46), and Greece (45), with fewer studies in Central and Eastern Europe (Fig. 2b). Research with a Europe-wide focus added 43–53 studies per country depending on the year of accession (43 studies for Romania and Bulgaria, EU27; 53 studies for EU15 countries; Fig. 2c). EU25 countries had a significantly lower number of regional, national, and multinational (Kruskal-Wallis *χ^2^_1_* =  10.106, p-value  = 0.0015), as well as total studies (*χ^2^_1_* = 11.447, p-value  = 0.0007) compared to EU15. The percent of national territory in N2K was weakly correlated with the number of total, and regional, national, and multinational studies (Spearman rank correlation *r_s_* = 0.05–0.56, with overall higher correlation for EU25 countries).

**Figure 3:**
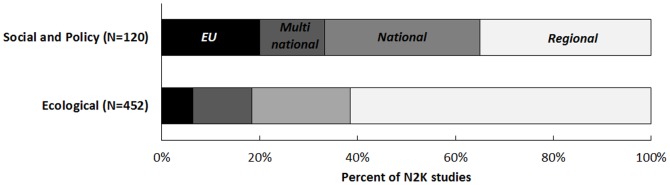
Percent of N2K studies by geographical scope.

‘Ecology’ studies were primarily focused on regional levels (one or more N2K sites within a country; 61.5%), and less on EU-wide extents (6.4%; Fig. 3). ‘Social and policy’ studies were conducted primarily at national and regional levels (66.6%), with EU-wide studies ranking higher relative to the ecological studies (20%; Fig. 3).

### Outcomes of N2K research

**Figure 4:**
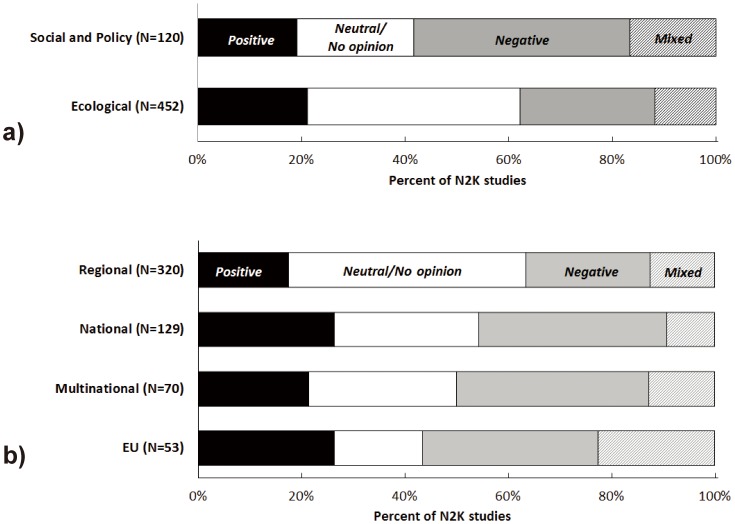
Conclusions of N2K studies by (a) study focus and (b) geographical scope.

Of the 572 studies, 360 (63.0%) reported positive, negative or mixed effects and/or opinions about N2K implementation; 168 studies found ‘ecological’ and ‘social and policy’ issues related to N2K implementation, ranging from inadequate EU policies and poor social acceptance (41.6% of ‘social and policy’ studies) to ineffective management and protection of biodiversity (26% of ‘ecological’ studies; Fig. 4a). A lower number of studies (N = 119, 20.8%) found positive effects of implementing N2K on biodiversity conservation and social participation; these studies mostly involved assessments of representation of specific taxa or habitats in N2K (see Table S1 for examples of study conclusions and outcomes). Negative effects of N2K were reported with relatively equal frequency at all scales of analysis (24.0–37.1%), while positive effects were reported most frequently at the national and EU levels (26.0% of studies in each category; Fig. 4b, Table S1).

### Network analysis of keywords

The 572 articles contained a total of 3433 keywords, of which 1692 are network nodes in the all-papers network (average number of keywords per article  = 5.99) (Table 1). The majority of keywords (74%, N = 1253) occur only once.

**Table 1:** Keyword and network-level metrics for the four keyword networks from Natura 2000 network (N2K) studies.

Network	# of papers	# of nodes	Average # of nodes per paper	Network density	Distance (shortest path length)	Network clustering	Random network distance	Random network clustering
All papers	572	1692	2.96	0.006	2.853	0.850	3.376	0.007
Ecology	452	1413	3.13	0.007	2.888	0.854	3.427	0.008
Social and policy	120	414	3.45	0.019	2.623	0.888	3.161	0.018

**Figure 5:**
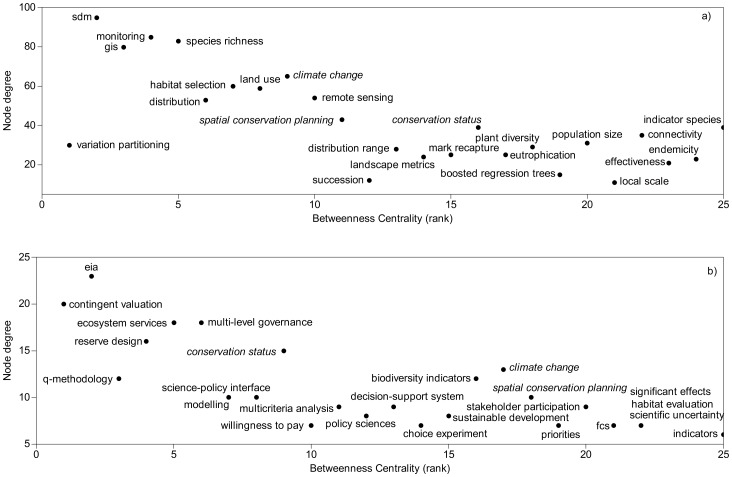
Important keywords describing methods used in a) ecology studies and b) social and policy studies. Keywords in italics are common for the two types of studies.

The keywords with highest degree and betweenness, which are therefore both important and critical in connecting other keywords, were the most general concepts: *Natura 2000, Habitats Directive, conservation, habitats, biodiversity, protected areas, Europe*, *nature conservation, and species distribution models* (Table 2). *Species distribution models* (SDM) had the highest degree of all keywords describing research methods (i.e., trendiest topic of N2K research), but low betweenness (i.e., does not link different areas of research). Other methods-specific keywords with moderate degree included analytical tools used for conservation planning and management such as *GIS, monitoring*, and *species richness*. Based on their betweenness, the methods keywords that link studies and areas of research were: *variation partitioning*, *SDM, GIS, monitoring, climate change, species richness, distribution, habitat selection*, and *land use* (Table 2); the same patterns were evident when ‘ecology’ and ‘social and policy’ articles were analyzed separately. For ‘ecology’ articles the most important methods keyword based on degree and betweenness are *species distribution models* (highest degree), *variation partitioning* (highest betweenness), *monitoring*, *GIS*, *species richness*, *distribution*, *habitat selection*, *land use*, etc. (Fig. 5a, Table S2a). ‘Policy and social’ studies focused on *environmental impact assessment, contingent valuation, ecosystem services, multi-level governance, agri-environment policy, reserve design*, etc. (Fig. 5b Table S2b). Important research keywords which are common for both articles types are *climate change*, *spatial conservation planning*, and *conservation status*.

**Table 2:** Important keywords emerging from all Natura 2000 network (N2K) studies (N = 572) published between 1996 and March 2014.

Keywords	Degree	Keywords	Betweenness
**Natura 2000**	984	**Natura 2000**	0.4170
**Habitats Directive**	679	**Habitats Directive**	0.2796
**conservation**	250	**conservation**	0.0584
**habitats**	198	**habitats**	0.0458
**biodiversity**	212	**biodiversity**	0.0435
**protected areas**	199	**protected areas**	0.0335
**EU**	143	**EU**	0.0284
**biodiversity conservation**	118	variation partitioning	0.0221
**sdm**	116	**nature conservation**	0.0213
**GIS**	98	**sdm**	0.0199
**nature conservation**	110	**biodiversity conservation**	0.0197
**monitoring**	102	**Spain**	0.0177
**species richness**	102	**Special Protection Area**	0.0174
**Spain**	89	**GIS**	0.0151
**climate change**	94	**monitoring**	0.0144
**Special Protection Area**	86	**climate change**	0.0142
**threatened species**	80	Euro-Siberian steppic woods	0.0138
management	76	**species richness**	0.0132
IUCN red list	81	distribution	0.0121
marine protected areas	81	**habitat selection**	0.0116
**habitat selection**	64	**wetlands**	0.0114
**wetlands**	69	**Water Framework Directive**	0.0112
**land use**	64	**Site of Community Importance**	0.0105
**Water Framework Directive**	69	**land use**	0.0103
**Site of Community Importance**	67	**threatened species**	0.0098

Keywords in bold have both high *degree* and high *betweenness*; *degree* measures the number of relations that run from a keyword to other keywords; *betweenness* measures the number of shortest paths that run through a keyword, and identifies keywords that connect other keywords (connectors).

**Figure 6:**
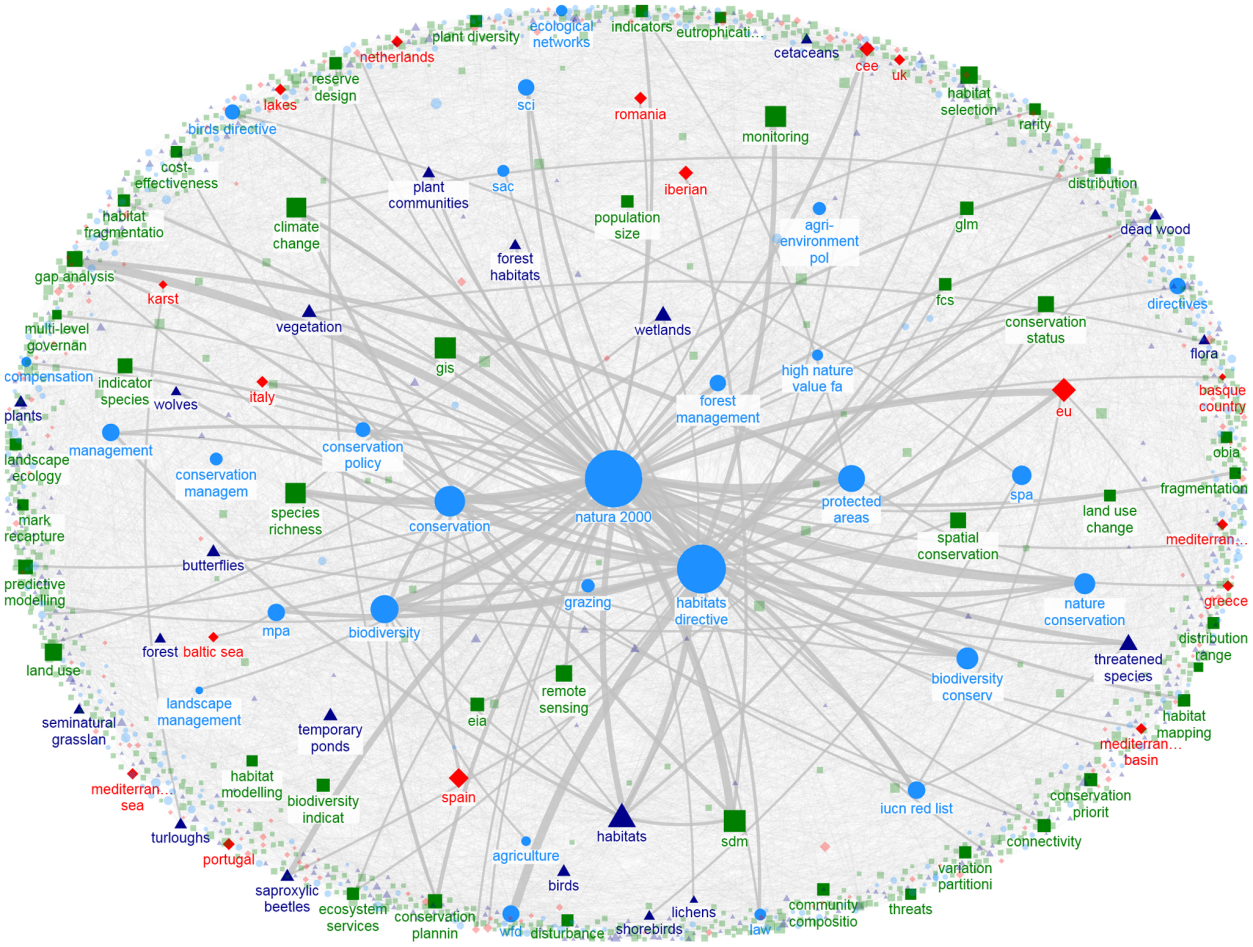
Keyword network for all N2K studies. Symbol size indicates node degree (maximum degree  = 656; only keywords with node degree>24 shown); Edge size (gray lines) indicates edge weights (maximum weight  = 24; only edges with weights>2 shown). Symbol color and shape indicates keyword category (light blue circles: management, green squares: methods, dark blue triangles: ecosystems/species, red diamonds: locations/institutions). Graph constructed using Fruchterman-Ringo layout with strength of repulsive force  = 100.

**Figure 7:**
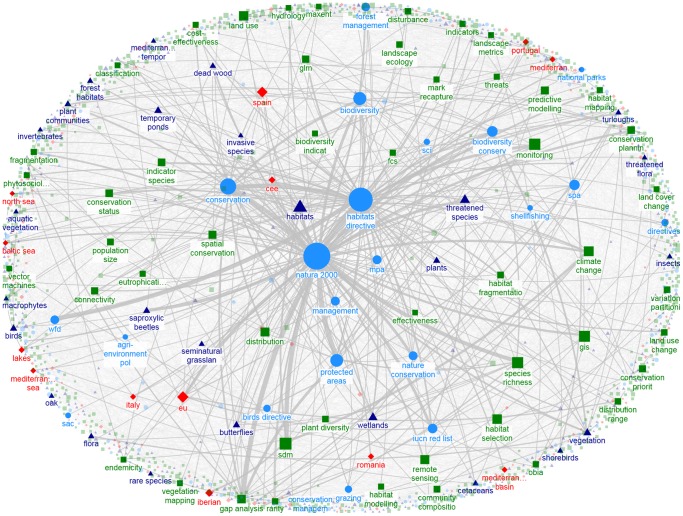
Keyword network for ecology studies; maximum node degree  = 489 (only keywords with node degree>19 shown); maximum edge weight  = 17 (only edges with weight>1 shown). Colors and symbols are similar to Figure 6.

**Figure 8:**
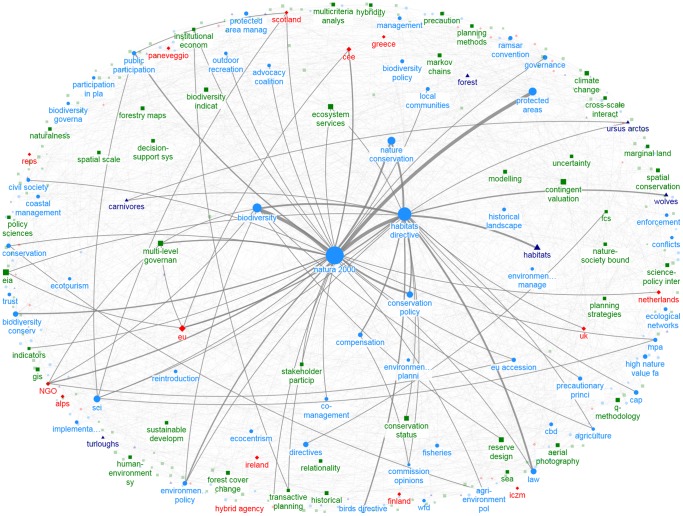
Keyword network for social and policy studies; maximum node degree  = 219 (only keywords with node degree>7 shown); maximum edge weight  = 7 (only edges with weight>1 shown). Colors and symbols are similar to Figure 6.

The distribution of degree centrality of keywords followed a power law, with a small number of highly connected keywords (i.e., maximum degree  = 984 for *Natura 2000*) and a majority of keywords with limited pairwise connections (median degree centrality  = 6). Thus, the N2K keyword network is a very low density network (0.006) with higher than expected clustering when compared to a random network (0.850 vs. 0.007, Table 2), conforming to the expectations of a “small-world” network. All papers are interconnected through an average of 2.85 keywords (shortest path length; Table 2), which together with density and clustering metrics implies that research themes are based on a set of closely-related concepts or keywords (Fig. 6). The same low-density network pattern with a few highly connected keywords emerges in the focus-specific networks (Table 2, Fig. 7, 8).

## Discussion

Our research showed that social and policy N2K research is under-represented, and that there is little correspondence between the ecological and social-policy fields. The prevalence of ecological research, largely dominated by conservation planning studies, came as no surprise, given that the implementation of the Habitats and Birds Directives is centered on conserving the species and habitats of Community importance [1]. Our application of network analysis of keywords demonstrated that N2K research is a small-world network, characteristic of real world phenomena, but also of well-developed fields of research [22], [23], [44]. This network property was still evident when ‘ecology’ and ‘social and policy’ research were analyzed separately. However, with the exception of key high-level concepts, such as *Natura 2000* or *Habitats Directive*, the two types of studies had a small number of other keywords in common, at low prevalence. This result highlights the fact that despite their apparent cohesion, research efforts have largely been singular within disciplines with little information shared across fields of study, and that N2K research may be lacking interdisciplinarity (even in countries where N2K has been addressed the most: Spain, Italy, and the UK).

### Past and current N2K research

We found an emphasis on ecological research in N2K (79% of studies), and particularly conservation planning. This finding can be interpreted as a direct result of the ongoing efforts to expand the N2K network [1], [9], but also a reflection of the low levels of penetration of social and political sciences in the field of conservation science [46], [47], [48]. Social sciences are integral to the discipline of conservation biology [19], yet the current conceptual, methodological, and epistemic gaps between the disciplines addressing ecology and society continue to hinder social and policy research in and for conservation [36]. These impediments, along with poor interdisciplinary communication [49], often result in conservation efforts that are disconnected from the social and political realities. In addition to the ecology/social science dichotomy, we found disparities in the distribution of research effort in Europe; the number of studies was biased toward the UK and countries in the Mediterranean basin, with little N2K research in Central, North, and Eastern Europe (Fig. 2b,c). Although we expected that regional, national and multinational N2K research to be more developed in countries with greater N2K coverage, we did not found evidence to substantiate this hypothesis. In addition, given the massive enlargement of N2K in recent years, especially in Central and Eastern European countries [1], [50], there may be a time lag between the designation of new N2K sites and the initiation of N2K research. Disparities in research effort among countries could also be explained by the ongoing development of institutional and management structures (e.g., N2K site administrations, management and conservation measures plans), as well as disparities in the availability of funding for conservation (e.g., between Western and Eastern Europe; [51]) or ability to publish in peer-reviewed journals [52].

Regional and national conservation targets can compromise EU-level objectives [53], and our findings on the outcomes and perceptions of N2K highlighted potential conflicts between national or regional and EU-level objectives for implementing the Habitats and Birds Directives. These conflicts manifested mostly at ‘social and policy’ (42% of studies) levels, likely driven by the fact that N2K expansion was not accompanied by national regulatory framework and proper community participation (e.g., [30]). At the same time, the negative conclusions of ‘social and policy’ studies reinforce the emerging view that social and institutional factors are important determinants of the efficiency of the N2K network [14], [54]. For example, a greater diversity of local conservation and social initiatives and community involvement can result in positive attitudes toward the natural and cultural heritage of traditional landscapes, and better conservation outcomes [14], [33], [34], [55]. Poor social acceptance of EU Directives, viewed as restrictions to land use, and the lack of stakeholder involvement in N2K site designation further highlight the disconnect between achieving conservation targets on paper and long-term sustainability [33].

More than 74% of keywords were unique to individual papers despite keyword standardization, suggesting that N2K research approaches and foci are extremely variable. This finding could be partially explained by factors not directly related to the topic of the papers; for example, taxon-specific research is highly fragmented among journals [9], which may have different keyword requirements (e.g., number of keywords, avoidance of words used in title). The distribution of keywords by degree and betweenness highlighted the most frequently addressed topics within each of the study types but also revealed critical concepts that link articles within fields of study. The ‘all-keyword’ and ‘ecology’ networks were dominated by keywords from the conservation planning realm describing analytical methods, which connected articles with a broad taxonomic, habitat type, and geographic extent. This is not surprising, since conservation planning is an important component of the conservation strategies that are mandatory for the managing N2K sites [56]. Keywords describing monitoring or adaptive management methods, which are critical components for N2K success [6] had low prevalence (monitoring: degree  = 102, betweenness  = 0.0144; Table 2). This finding was expected given that N2K is still in its infancy in many countries, and many sites are still at the management planning stage.

*Multi-level governance* and *ecosystem services* were among key topics in the ‘social and policy’ studies keyword network. Multi-level governance is a political concept developed in Europe in the 1990s that refers to interacting authority structures, and emphasizes the dispersion of decision-making from local to continental or global levels [57]. This topic is highly relevant to N2K because of the need to link local policy and decision-making (implementation, monitoring) to national (enforcement, spatial planning) and EU (red lists) policies [6], and its prevalence in the N2K literature highlights an emerging area of N2K research. The other key topic of social and policy studies, *ecosystem services*, highlights an area of research which is gaining prominence in N2K research, as this concept provides the medium for integrating social and economic aspects into conservation decisions, and informing spatial conservation planning [58], [59], [60], [61].

Our study used network analysis to provide a broad perspective on N2K research, encompassing the full breadth of research approaches and concepts, but at the detriment of depth. While this is a potential drawback, we consider this study to be a first step towards a better understanding of the trends and limitations of existing approaches to N2K research. Deeper insight into particular aspects of N2K research could be gained by using meta-analysis or systematic reviews ([62]; e.g., [9], [61]), but this was beyond the scope of this study. For example, future evidence syntheses of N2K research could focus on the evaluating emerging patterns in the social acceptance of N2K, on the conflicts generated by the implementation of sectoral policies, etc.

### Prospects for future of N2K research

As with every conservation initiative, the efficacy of N2K depends highly on the knowledge of social-ecological system, which both guides and is guided by the vision and expected outcomes of N2K implementation [63]. Even though the majority of N2K sites were created as tightly coupled social-ecological systems, research addressing the nature of multiple links between the society and the landscape, and their consequences on the whole social-ecological system are largely missing. Most current research focuses on describing biodiversity patterns within the N2K sites, thus targeting only one of the outcomes of the social-ecological interactions. Despite the almost exclusive focus on the ecology of N2K, such information does not percolate into designing or updating N2K management plans [9], further highlighting the dichotomy between academic research and conservation practice [64].

While ecological studies are fundamental for science-based conservation in N2K, we believe that N2K research must reach well beyond prioritizing areas for conservation of taxa and habitats of EU interest. Although understanding these ecological aspects is of crucial importance to N2K management, this research should be embedded in the social-ecological reality of the N2K. Such an understanding can be provided by inter- and transdisciplinary research agendas [65], [66], which can generate and integrate information about ecology, ecosystem services, and institutions, along with their historical interactions. We agree with Hochkirch and colleagues [6] that fundamental changes are needed in the way the Habitats and Birds Directives are implemented, and that past and current research has largely ignored an approach in which conservation targets would be addressed simultaneously with national and EU environmental policies, and social systems.

New research should address emerging conflicts between development and conservation priorities in N2K, which are partly fueled by conflicting sectoral policies and EU funding frameworks (e.g., between EU agricultural reform and conservation [67], or between renewable energy development and conservation [68]). Practical solutions for alleviating such conflicts require an understanding of the tradeoffs between social and economic incentives and the opportunities for biodiversity conservation. Another critical step forward should be dissemination of EU funded conservation project findings in the peer-reviewed literature. For example, LIFE Nature, the main EU financial instrument for N2K, which in the past 20 years co-financed more than 4,000 projects across the EU member states, has no specific requirements for the dissemination of findings in the peer-reviewed literature (http://ec.europa.eu/environment/life/about/index.htm). We found few studies reporting the results of LIFE Nature projects, highlighting again the dichotomy between conservation practice and science. Moreover, we encourage the regular reporting of monitoring results in the peer-reviewed literature, which is important for coordinating conservation efforts across N2K sites [9]. In addition to financing tools targeted at such research, conservation, ecological and social science journals can play equally important roles in stimulating social-ecological N2K research (e.g., special issues on integrative social, policy, and conservation research in N2K).

### Summary

Our study showed that most of the research conducted on N2K is reactive rather than proactive, focuses on the ecological systems, and lacks a holistic vision that integrates society and ecological systems. Because many of the EU regions covered by N2K regulations are cultural landscapes, future research addressing tradeoffs between economic targets, social desires, and biodiversity conservation in N2K is urgently needed. In order to be relevant for the management of N2K sites, academic research should be integrative across fields of study, conducted in close collaboration with nature protection agencies, and more effectively disseminated outside academia [9], [49]. Developing an N2K research agenda that evaluates and identifies opportunities for nature protection and society, ecologists must expand their “small world” network, and match the social-ecological perspective for biodiversity conservation [69].

## Supporting Information

Figure S1 **Percent of national territory in terrestral Natura 2000 sites for EU27 countries.** [AT  =  Austria, BE  =  Belgium, BG  =  Bulgaria, CY  =  Cyprus, CZ  =  Czech Republic, DE  =  Germany, DK  =  Denmark, EE  =  Estonia, EL  =  Greece, ES  =  Spain, FI  =  Finland, FR  =  France, IE  =  Ireland, IT  =  Italy, LT  =  Lithuania, LU  =  Luxembourg, LV  =  Latvia, MT  =  Malta, NL  =  Netherlands, PL  =  Poland, PT  =  Portugal, RO  =  Romania, SE  =  Sweden, SI  =  Slovenia, SK  =  Slovakia, UK  =  United Kingdom].
(TIF)

Table S1 **Examples of negative and positive conclusions in N2K research.** See Data S1 for references.
(DOCX)

Table S2 **Important keywords emerging from Natura 2000 network papers with a) ecology focus (N = 452) and b) social and policies focus (N = 120), published between 1996 and March 2014.** Keywords in bold have both high degree and high betweenness; degree measures the number of relations that run from a keyword to other keywords; betweenness measures the number of shortest paths that run through a keyword, and identifies keywords that connect other keywords (connectors).
(DOCX)

File S1 **Extended definitions of network metrics.**
(DOCX)

Checklist S1 **PRISMA 2009 checklist.**
(DOC)

Data S1 **Natura 2000 literature reviewed in this study.**
(DOCX)

Data S2 **Raw data used to conduct analyses (MS Excel file).**
(XLS)

Data S3 **Peer-reviewed papers and conference proceedings reviewed in this study in BibTeX format (for import into reference management software).**
(TXT)
