# Supplementary figures and images for: Species, Habitats, Society: An Evaluation of Research Supporting EU's Natura 2000 Network
Source: PLoS One. 2014 Nov 21;9(11):e113648. doi: 10.1371/journal.pone.0113648 (PMC4240592; doi:10.1371/journal.pone.0113648)

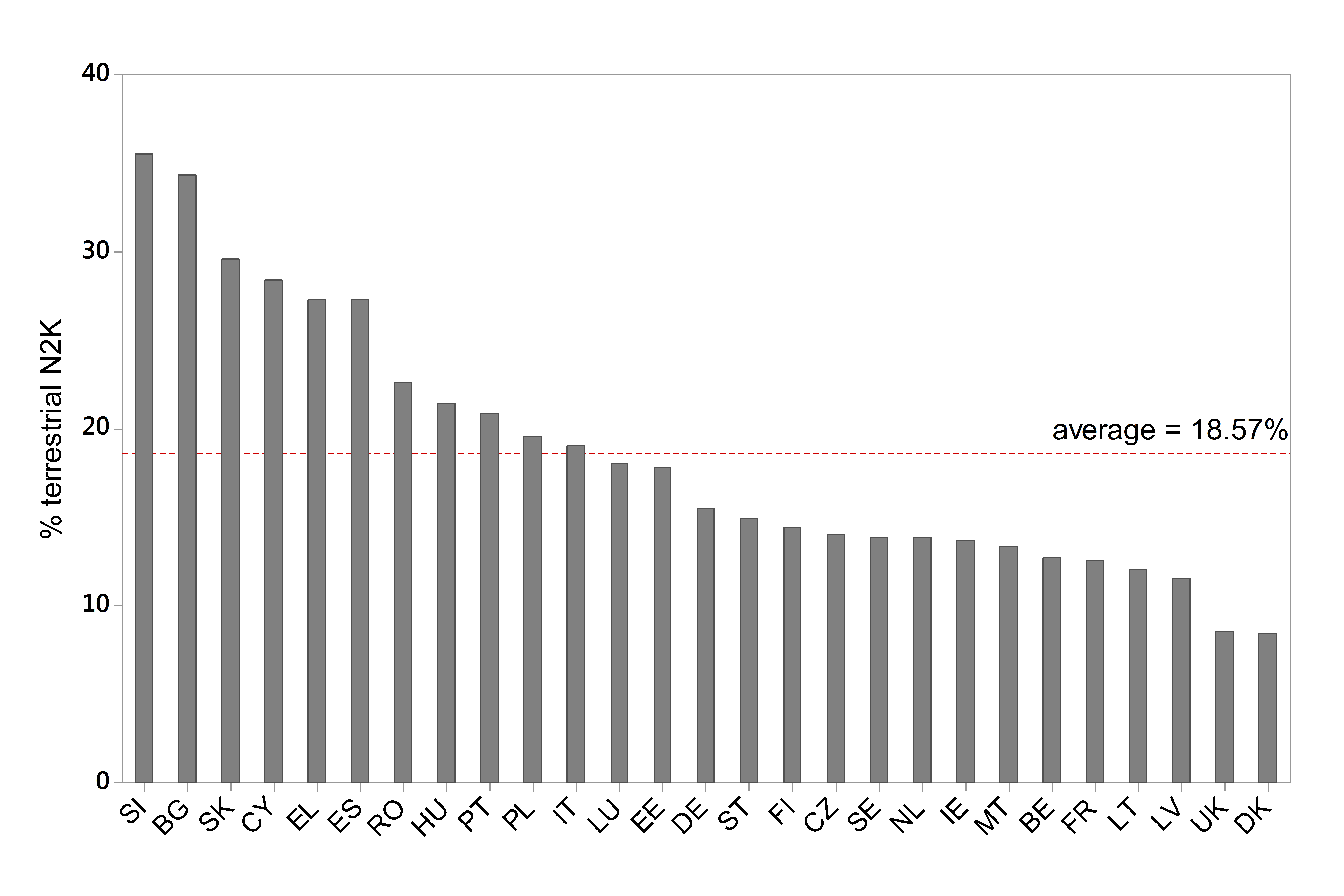

Supplement: Figure S1 — Percent of national territory in terrestral Natura 2000 sites for EU27 countries. [AT = Austria, BE = Belgium, BG = Bulgaria, CY = Cyprus, CZ = Czech Republic, DE = Germany, DK = Denmark, EE = Estonia, EL = Greece, ES = Spain, FI = Finland, FR = France, IE = Ireland, IT = Italy, LT = Lithuania, LU = Luxembourg, LV = Latvia, MT = Malta, NL = Netherlands, PL = Poland, PT = Portugal, RO = Romania, SE = Sweden, SI = Slovenia, SK = Slovakia, UK = United Kingdom]. (TIF) [file pone.0113648.s001.tif]
