# Supplementary material for: Species, Habitats, Society: An Evaluation of Research Supporting EU's Natura 2000 Network
Source: PLoS One. 2014 Nov 21;9(11):e113648. doi: 10.1371/journal.pone.0113648 (PMC4240592; doi:10.1371/journal.pone.0113648)
Supplement: Table S1 — Examples of negative and positive conclusions in N2K research. See Data S1 for references. (DOCX) [file pone.0113648.s002.docx]

**Table S1.** Examples of negative and positive conclusions in N2K research. See Data S1 for references

| **Negative outcome** | **Study findings** | |
| --- | --- | --- |
| Efficacy | Lack of common standards for Favourable Conservation Status and monitoring (Abellan et al. 2007; Branquart et al. 2008; Brown 2000; Ganatsas et al. 2013; Hernando et al. 2010; Li et al. 2004; Panitsa et al. 2011) | |
|  | There are many species in Annex II of Habitats Directive, but not always the right ones for Spain (Lozano et al. 1996) | |
|  | Endangered mollusks not protected by Habitats Directive, some protected species need to be delisted (Bouchet et al. 1999; Katsanevakis et al. 2011) | |
|  | Some habitats wrongly described by Habitats Directive for Atlantic and Mediterranean beach and foredunes (Feola et al. 2011) | |
|  | Shrub-steppe fragments and invertebrates unprotected in Spain (Hernandez-Manrique et al. 2012; Laiolo and Tella 2006) | |
|  | Italian N2K network not effective for most species, including amphibians (D'Amen et al. 2011; [Maiorano et al. 2007](#_ENREF_12)) | |
|  | N2K not enough extended in Crete, Ioanian Sea and its shore, lower elevation and woodland habitats in UK, mires in Bulgaria (Dimitrakopoulos et al. 2004; Giakoumi et al. 2012; Hajek M. et al. 2010; Jackson and Gaston 2008) | |
|  | N2K sites has been superimposed upon the existing protected areas in Greece and Romania (Papageorgiou and Vogiatzakis 2006; Ioja et al. 2010) | |
|  | Farmland species are systematically excluded by protected area, including N2K (Mendoza-Fernandez et al. 2010) | |
|  | The connectivity of N2K sites among EU country borders are weak (Opermanis et al. 2012)  N2K fail to adequate represent areas of lower elevation and woodland habitats (Jackson and Gaston 2008) | |
| Inadequate policy | In the case of priority species (e.g. brown bears) each state interpret the HD, thus, the species are not unitary protected (Rosen and Bath 2009) | |
|  | Conservation terms are vaguely defined in DH (Louette et al. 2011)  N2K implementation led to fast de-institutionalization of nature protection in NL (Beunen et al. 2013) but reinforce the central bureaucracy in CZ (Prazan et al. 2005) | |
|  | EU policies on Agriculture, Fishing, and Nature protection are not correlated (De Santo and Jones 2007; Litskas et al. 2013) | |
|  | Explicit quantitative goals for conservation planning have largely been missing (Gaston et al. 2008) | |
|  | The EU no biodiversity net-loss policy is ineffective (Ledoux et al. 2000) | |
|  | The implementation of HD is behind the schedule in France. Implementation of N2K it is an example of the way the society reflects on the treatment of the environment (Alphandery and Fortier 2010)  Policies supporting farmland biodiversity in FI are constrained by inflexible EU regulations (Arponen et al. 2013) | |
| Poor social acceptance | N2K is often perceived by rural landholders as a new form of “colonialism” in Ireland (Bryan 2012) | |
|  | People are against protection in Finland and Portugal (Li et al. 2004) | |
|  | N2K is perceived as an unnecessary additional conservation tool by both local authorities and communities residing in the N2K areas in Poland (Grodzinska-Jurczak and Cent 2011) | |
|  | The European and national administrations keep discussion on European habitats and their partial or complete impairment non-public (Kramer 2009) | |
| Ineffective management | Conservation cost-effectiveness is not assessed or of minor relevance on management plans (Watzold et al. 2010; Watzold and Schwerdtner 2005)  Habitats monitoring activities are fragmented even at regional level (Lengyel et al. 2008)  The data for management actions are generally scarce (Ganatsas et al. 2013) | |
|  | Conflicts between N2K and renewable energy sector in Poland (Jackson 2011) | |
|  | Lack of financial compensations for the landowners in Romania (Stancioiu et al. 2010) | |
| Positive outcome | European Court of Justice interpret the term priority very restrictive, in a good way (Miller 1997)  N2K effective for conserving agri-ecosystems (Beaufoy 1998)  Low intensive agriculture is beneficial for habitats (Ostermann 1998)  Good protection assured by MPAs in Mediterranean area (Sanchez-Fernandez et al. 2004)  Iberian N2K reasonably large, effective for flora of eastern Andalusia and for lichens in Spain (Araujo et al. 2007; Mendoza-Fernandez et al. 2010; Martinez et al. 2006)  PAs include high density of common declining species (Devictor et al. 2007)  Italian N2K coherent for habitats (Rosati et al. 2008)  Estonia has achieved good results in wetland protection (Kimmel et al. 2010)  Forests habitats in Slovenia are in favourable conservation status and N2K is effective for butterflies (Kutnar et al. 2011; Verovnik et al. 2011)  Habitats Directive slowed down some harbor capacity upgrade (Morris 2011)  NGOs are critical stakeholder in N2K implementation in Poland and Hungary (Cent et al. 2013) |  |
|  |  |  |
