# Supplementary material for: Species, Habitats, Society: An Evaluation of Research Supporting EU's Natura 2000 Network
Source: PLoS One. 2014 Nov 21;9(11):e113648. doi: 10.1371/journal.pone.0113648 (PMC4240592; doi:10.1371/journal.pone.0113648)
Supplement: Table S2 — Important keywords emerging from Natura 2000 network papers with a) ecology focus (N = 452) and b) social and policies focus (N = 120), published between 1996 and March 2014. Keywords in bold have both high degree and high betweenness; degree measures the number of relations that run from a keyword to other keywords; betweenness measures the number of shortest paths that run through a keyword, and identifies keywords that connect other keywords (connectors). (DOCX) [file pone.0113648.s003.docx]

**Table S2**. Important keywords emerging from Natura 2000 network papers with a) ecology focus (N=452) and b) social and policies focus (N=120), published between 1996 and March 2014. Keywords in bold have both high *degree* and high *betweenness*; *degree* measures the number of relations that run from a keyword to other keywords; *betweenness* measures the number of shortest paths that run through a keyword, and identifies keywords that connect other keywords (connectors).

1. Ecology papers

| **Keywords** | **Degree** | **Keywords** | **Betweenness** |
| --- | --- | --- | --- |
| **Natura 2000** | 493 | **Natura 2000** | 0.3746 |
| **Habitats Directive** | 397 | **Habitats Directive** | 0.2806 |
| **conservation** | 176 | **conservation** | 0.0711 |
| **habitats** | 135 | **habitats** | 0.0502 |
| **biodiversity** | 120 | **biodiversity** | 0.0414 |
| **protected areas** | 110 | **EU** | 0.0309 |
| **sdm** | 95 | **protected areas** | 0.0308 |
| **EU** | 89 | variation partitioning | 0.0262 |
| **monitoring** | 85 | **sdm** | 0.0259 |
| **species richness** | 83 | **Spain** | 0.0224 |
| **GIS** | 80 | **GIS** | 0.0192 |
| **Spain** | 78 | **monitoring** | 0.0189 |
| **biodiversity conservation** | 77 | **Special Protection Area** | 0.0188 |
| **Special Protection Area** | 73 | **biodiversity conservation** | 0.0180 |
| **threatened species** | 69 | **species richness** | 0.0178 |
| **climate change** | 65 | euro-siberian steppic woods | 0.0164 |
| IUCN red list | 62 | **nature conservation** | 0.0164 |
| **habitat selection** | 60 | **distribution** | 0.0150 |
| **land use** | 59 | **habitat selection** | 0.0149 |
| **wetlands** | 59 | **land use** | 0.0144 |
| **nature conservation** | 57 | **wetlands** | 0.0143 |
| Marine Protected Areas | 54 | **threatened species** | 0.0140 |
| remote sensing | 54 | **climate change** | 0.0129 |
| management | 54 | Water Framework Directive | 0.0126 |
| **distribution** | 53 | Iberian peninsula | 0.0119 |

1. Social and policy papers

| **Keywords** | **Degree** | **Keywords** | **Betweenness** |
| --- | --- | --- | --- |
| **Natura 2000** | 219 | **Natura 2000** | 0.5678 |
| **Habitats Directive** | 124 | **Habitats Directive** | 0.2876 |
| **biodiversity** | 55 | **biodiversity** | 0.0575 |
| **protected areas** | 43 | **nature conservation** | 0.0461 |
| **nature conservation** | 42 | **Site of Community Importance** | 0.0365 |
| **Site of Community Importance** | 32 | **contingent valuation** | 0.0338 |
| **habitats** | 31 | **protected areas** | 0.0314 |
| **EU** | 31 | **EU** | 0.0302 |
| **conservation policy** | 28 | trust | 0.0275 |
| **law** | 24 | **habitats** | 0.0259 |
| **EIA** | 23 | **EIA** | 0.0254 |
| **directives** | 20 | **directives** | 0.0245 |
| **biodiversity conservation** | 20 | **law** | 0.0203 |
| **contingent valuation** | 20 | q-methodology | 0.0184 |
| **ecosystem services** | 18 | **reserve design** | 0.0180 |
| **multi-level governance** | 18 | **biodiversity conservation** | 0.0153 |
| **agri-environment policy** | 17 | **conservation policy** | 0.0149 |
| CEE | 17 | **agri-environment policy** | 0.0147 |
| **reserve design** | 16 | **ecosystem services** | 0.0120 |
| environmental policy | 15 | fisheries | 0.0091 |
| Common Agriculture Policiy | 15 | ICZM | 0.0087 |
| **compensation** | 15 | CBD | 0.0087 |
| conservation status | 15 | **compensation** | 0.0077 |
| **governance** | 15 | **multi-level governance** | 0.0064 |
| conservation | 14 | **governance** | 0.0061 |
