# Supplementary material for: Species, Habitats, Society: An Evaluation of Research Supporting EU's Natura 2000 Network
Source: PLoS One. 2014 Nov 21;9(11):e113648. doi: 10.1371/journal.pone.0113648 (PMC4240592; doi:10.1371/journal.pone.0113648)
Supplement: File S1 — Extended definitions of network metrics. (DOCX) [file pone.0113648.s004.docx]

**File S1. Extended definitions of network metrics used in this manuscript**

*Keywords standardization*

Because particular keywords might appear under a slight different expression across articles but have the same meaning, we standardized such keywords (i.e., by converting from singular or plural form [*Habitat Directive* to *Habitats Directive*]; choosing the abbreviated or unabbreviated form [*Central and Eastern European countries* to *CEE*]; removing hyphens [*Discrete-choice model* to *Discrete choice model*]; removing articles [*The Netherlands* to *Netherlands*]; unifying synonyms [*Conservation biology* to *Biodiversity conservation*]).

*Networks metrics definitions*

The *node degree* is equal to the number of *edges* (pairs of keywords) connected with the respective node, and may be normalised as *degree centrality*. A *node* with a high *degree centrality* has many connections in the network, and may suggest that it connects papers that share the same ideas, or approach different topics using similar methods. The more connected a keyword, the more central is the keyword in the network, indicating high importance in N2K research. The *betweenness centrality* of a *node* measures the degree to which a node lies on the shortest path between other nodes. *Betweenness centrality* for a keyword A is measured as the number of *shortest paths* that pass through a node, divided by all *shortest paths* in the network. The metric is useful to identify the keywords that act as links and connect otherwise disparate keywords. Such keywords may have a lesser *degree centrality*, but they act as connectors between subfields or types of studies.

The *keyword-to-keyword distance* (or *shortest path*) is defined as the minimum number of keywords traversed to get from keyword A to keyword B (i.e., geodesic distance). The average *shortest path* is calculated at the network level as the average number of shortest steps of all possible pairs of the network *nodes*. The measure gives a sense of how close are the keywords in the network. A large average *shortest path* suggests the existence of many disconnected keywords in the respective network, and thus low similarity between the papers. The metric is useful for comparing networks (e.g. conservation planning vs. conservation policy), and the flow of methods or ideas tends to be more interrelated in the networks with the lower average keyword-to-keyword distance.

The *network density* expresses the proportion of *edges* (pairs of keywords) out of the total number of possible *edges* (e.g., between all possible keyword pairs). The *network density* takes value between 0 (only independent keyword in the network and no *edges*) and 1 (clique-type network, where all keywords are interconnected). Because the number of connections in a network grow logarithmically with the size of the network, the metric is useful for when comparing networks against each other, or subsets of networks. The *clustering coefficient* of a *node* is calculated at network level as the ratio of number of connections between the all neighbors of the node to all possible connections between neighbors. For example, assume that the keyword “Natura 2000” is connected with neighbors “CEE”, “Efficacy” and “Birds”. If only one connection exist between the three neighbors (e.g., CEE-to-Birds), then the clustering coefficient is 0.33.
