# Supplementary material for: Species, Habitats, Society: An Evaluation of Research Supporting EU's Natura 2000 Network
Source: PLoS One. 2014 Nov 21;9(11):e113648. doi: 10.1371/journal.pone.0113648 (PMC4240592; doi:10.1371/journal.pone.0113648)
Supplement: Data S1 — Natura 2000 literature reviewed in this study. (DOCX) [file pone.0113648.s006.docx]

**Data S1. Natura 2000 literature reviewed in this study**

1. Abbiati M, Mistri M, Bartoli M, Ceccherelli VU, Colangelo MA, et al. (2010) Trade-off between conservation and exploitation of the transitional water ecosystems of the northern Adriatic Sea. Chemistry and Ecology 26: 105-119.

2. Abellan MD, Martinez JE, Palazon JA, Esteve MA, Calvo JF (2011) Efficiency of a Protected-Area Network in a Mediterranean Region: A Multispecies Assessment with Raptors. Environmental Management 47: 983-991.

3. Abellan P, Sanchez-Fernandez D, Velasco J, Millan A (2007) Effectiveness of protected area networks in representing freshwater biodiversity: the case of a Mediterranean river basin (south-eastern Spain). Aquatic Conservation-Marine and Freshwater Ecosystems 17: 361-374.

4. Alagador D, Trivino M, Cerdeira JO, Bras R, Cabeza M, et al. (2012) Linking like with like: optimising connectivity between environmentally-similar habitats. Landscape Ecology 27: 291-301.

5. Albert J, Platek M, Cizek L (2012) Vertical stratification and microhabitat selection by the Great Capricorn Beetle (Cerambyx cerdo) (Coleoptera: Cerambycidae) in open-grown, veteran oaks. European Journal of Entomology 109: 553-559.

6. Albuquerque FS, Assunção-Albuquerque MJT, Cayuela L, Zamora R, Benito BM (2013) European Bird distribution is “well” represented by Special Protected Areas: Mission accomplished? Biological Conservation 159: 45-50.

7. Alexandridis TK, Lazaridou E, Tsirika A, Zalidis GC (2009) Using Earth Observation to update a Natura 2000 habitat map for a wetland in Greece. Journal of Environmental Management 90: 2243-2251.

8. Ali I, Schuster C, Zebisch M, Forster M, Kleinschmit B, et al. (2013) First Results of Monitoring Nature Conservation Sites in Alpine Region by Using Very High Resolution (VHR) X-Band SAR Data. Ieee Journal of Selected Topics in Applied Earth Observations and Remote Sensing 6: 2265-2274.

9. Alphandery P, Fortier A (2010) Local Settings and Biodiversity A Sociological Approach to the Implementation of the EC Habitats Directive in France. Current Sociology 58: 755-776.

10. Amici V, Santi E, Filibeck G, Diekmann M, Geri F, et al. (2013) Influence of secondary forest succession on plant diversity patterns in a Mediterranean landscape. Journal of Biogeography 40: 2335-2347.

11. Anadon JD, Gimenez A, Perez I, Martinez M, Esteve MA (2006) Habitat selection by the spur-thighed tortoise Testudo graeca in a multisuccessional landscape: Implications for habitat management. Biodiversity and Conservation 15: 2287-2299.

12. Andersen DK, Nygaard B, Fredshavn JR, Ejrnaes R (2013) Cost-effective assessment of conservation status of fens. Applied Vegetation Science 16: 491-501.

13. Anderwald P, Haberlin MD, Coleman M, Cadhla OO, Englund A, et al. (2012) Seasonal trends and spatial differences in marine mammal occurrence in Broadhaven Bay, north-west Ireland. Journal of the Marine Biological Association of the United Kingdom 92: 1757-1766.

14. Andreou M, Delipetrou P, Kadis C, Tsiamis G, Bourtzis K, et al. (2011) An integrated approach for the conservation of threatened plants: The case of Arabis kennedyae (Brassicaceae). Acta Oecologica-International Journal of Ecology 37: 239-248.

15. Andrulewicz E, Otremba Z, Kaminska K (2010) Ongoing Technical Activities and Conservation Measures in Maritime Spatial Planning within Polish Marine Areas. Polish Journal of Environmental Studies 19: 553-563.

16. Andueza IL, Alcayde VS (2004) Advances in the action plan for Emys orbicularis in the Valencia region, Spain. Biologia 59: 173-176.

17. Anthon S, Garcia S, Stenger A (2010) Incentive Contracts for Natura 2000 Implementation in Forest Areas. Environmental & Resource Economics 46: 281-302.

18. Apostolopoulou E, Drakou EG, Pediaditi K (2012) Participation in the management of Greek Natura 2000 sites: evidence from a cross-level analysis. J Environ Manage 113: 308-318.

19. Apostolopoulou E, Pantis JD (2009) Conceptual gaps in the national strategy for the implementation of the European Natura 2000 conservation policy in Greece. Biological Conservation 142: 221-237.

20. Araujo MB (2004) Matching species with reserves - uncertainties from using data at different resolutions. Biological Conservation 118: 533-538.

21. Araujo MB, Alagador D, Cabeza M, Nogues-Bravo D, Thuiller W (2011) Climate change threatens European conservation areas. Ecology Letters 14: 484-492.

22. Araujo MB, Lobo JM, Moreno JC (2007) The effectiveness of Iberian protected areas in conserving terrestrial biodiversity. Conservation Biology 21: 1423-1432.

23. Arconada B, Delgado P, Garcia A (2013) Minimizing environmental risks on constructing marine pipelines: Aguilas desalination plant. Desalination and Water Treatment 51: 246-261.

24. Arponen A, Heikkinen RK, Paloniemi R, Poyry J, Simila J, et al. (2013) Improving conservation planning for semi-natural grasslands: Integrating connectivity into agri-environment schemes. Biological Conservation 160: 234-241.

25. Arts K, Fischer A, van der Wal R (2014) Political decision making, governance shifts and Scottish animal reintroductions: are democratic principles at stake? Journal of Environmental Planning and Management 57: 612-628.

26. Assini S, Mondino GP, Varese P, Barcella M, Bracco F (2013) A phytosociological survey of the Corynephorus canescens (L.) Beauv. communities of Italy. Plant Biosystems 147: 64-78.

27. Atalah J, Fitch J, Coughlan J, Chopelet J, Coscia I, et al. (2013) Diversity of demersal and megafaunal assemblages inhabiting sandbanks of the Irish Sea. Marine Biodiversity 43: 121-132.

28. Atkinson PW, Maclean IM, Clark NA (2010) Impacts of shellfisheries and nutrient inputs on waterbird communities in the Wash, England. Journal of Applied Ecology 47: 191-199.

29. Azzella MM, Iberite M, Fascetti S, Rosati L (2013) Loss detection of aquatic habitats in Italian volcanic lakes using historical data. Plant Biosystems 147: 521-524.

30. Baattrup-Pedersen A, Andersen HE, Larsen SE, Nygaard B, Ejrnaes R (2012) Predictive modelling of protected habitats in riparian areas from catchment characteristics. Ecological Indicators 18: 227-235.

31. Baattrup-Pedersen A, Larsen SE, Mejlhede P, Audet J, Hoffman CC, et al. (2011) Stream characteristics and their implications for the protection of riparian fens and meadows. Freshwater Biology 56: 1893-1903.

32. Bacaro G, Santi E, Rocchini D, Pezzo F, Puglisi L, et al. (2011) Geostatistical modelling of regional bird species richness: exploring environmental proxies for conservation purpose. Biodiversity and Conservation 20: 1677-1694.

33. Bacchetta G, Farris E, Pontecorvo C (2012) A new method to set conservation priorities in biodiversity hotspots. Plant Biosystems 146: 638-648.

34. Back S, Ekebom J, Kangas P (2002) A proposal for a long-term baseline phytobenthos monitoring programme for the Finnish Baltic coastal waters: Monitoring submerged rocky shore vegetation. Environmental Monitoring and Assessment 79: 13-27.

35. Bagella S, Caria MC, Farris E, Filigheddu R (2009) Spatial-time variability and conservation relevance of plant communities in Mediterranean temporary wet habitats: A case study in Sardinia (Italy). Plant Biosystems 143: 435-442.

36. Bagella S, Caria MC, Filigheddu R (2013) Gap analysis revealed a low efficiency of Natura 2000 network for the conservation of endemic species in Mediterranean temporary freshwater habitats. Plant Biosystems 147: 1092-1094.

37. Bagella S, Caria MC, Zuccarello V (2010) Patterns of emblematic habitat types in Mediterranean temporary wetlands. Comptes Rendus Biologies 333: 694-700.

38. Barbera C, Moranta J, Ordines F, Ramon M, de Mesa A, et al. (2012) Biodiversity and habitat mapping of Menorca Channel (western Mediterranean): implications for conservation. Biodiversity and Conservation 21: 701-728.

39. Barberán R, Egea P, Pérez LP (2005) The cost of Natura 2000 in Spain. European Environment 15: 161-174.

40. Barbier Y, Lejeune P, Dufrene M, Rondeux J (2009) An ArcGIS-based tool for the relational data management assistance of Natura 2000 habitat units in the Walloon Region, Belgium. Biotechnologie Agronomie Societe Et Environnement 13: 243-248.

41. Bassler C, Stadler J, Muller J, Forster B, Gottlein A, et al. (2011) LiDAR as a rapid tool to predict forest habitat types in Natura 2000 networks. Biodiversity and Conservation 20: 465-481.

42. Bastian O (2013) The role of biodiversity in supporting ecosystem services in Natura 2000 sites. Ecological Indicators 24: 12-22.

43. Beaufoy G (1998) The EU Habitats Directive in Spain: can it contribute effectively to the conservation of extensive agroecosystems? Journal of Applied Ecology 35: 974-978.

44. Bekkby T, Isaeus M (2008) Mapping large, shallow inlets and bays: modelling a Natura 2000 habitat with digital terrain and wave-exposure models. Ices Journal of Marine Science 65: 238-241.

45. Benesperi R, Giuliani C, Zanetti S, Gennai M, Lippi MM, et al. (2012) Forest plant diversity is threatened by Robinia pseudoacacia (black-locust) invasion. Biodiversity and Conservation 21: 3555-3568.

46. Benesperi R, Lastrucci L, Nascimbene J (2013) Human Disturbance Threats the Red-Listed Macrolichen Seirophora villosa (Ach.) Frod,n in Coastal Juniperus Habitats: Evidence From Western Peninsular Italy. Environmental Management 52: 939-945.

47. Berces S, Elek Z (2013) Overlapping generations can balance the fluctuations in the activity patterns of an endangered ground beetle species: long-term monitoring of Carabus hungaricus in Hungary. Insect Conservation and Diversity 6: 290-299.

48. Bergmeier E, Petermann J, Schroder E (2010) Geobotanical survey of wood-pasture habitats in Europe: diversity, threats and conservation. Biodiversity and Conservation 19: 2995-3014.

49. Beunen R, de Vries JR (2011) The governance of Natura 2000 sites: the importance of initial choices in the organisation of planning processes. Journal of Environmental Planning and Management 54: 1041-1059.

50. Beunen R, van Assche K (2013) Contested delineations: planning, law, and the governance of protected areas. Environment and Planning A 45: 1285-1301.

51. Beunen R, Van Assche K, Duineveld M (2013) Performing failure in conservation policy: The implementation of European Union directives in the Netherlands. Land Use Policy 31: 280-288.

52. Biondi E, Casavecchia S, Pesaresi S (2010) Interpretation and management of the forest habitats of the Italian peninsula. Acta Botanica Gallica 157: 687-719.

53. Biondi E, Casavecchia S, Pesaresi S, Zivkovic L (2012) Natura 2000 and the Pan-European Ecological Network: a new methodology for data integration. Biodiversity and Conservation 21: 1741-1754.

54. Biondi E, Zivkovic L, Esposito L, Pesaresi S (2009) Vegetation, plant landscape and habitat analyses of a fluvial ecosystem in central Italy. Acta Botanica Gallica 156: 571-587.

55. Bittner T, Jaeschke A, Reineking B, Beierkuhnlein C (2011) Comparing modelling approaches at two levels of biological organisation - Climate change impacts on selected Natura 2000 habitats. Journal of Vegetation Science 22: 699-710.

56. Blasi C, Marignani M, Copiz R, Fipaldini M, Bonacquisti S, et al. (2011) Important Plant Areas in Italy: From data to mapping. Biological Conservation 144: 220-226.

57. Bock M, Rossner G, Wissen M, Remm K, Langanke T, et al. (2005) Spatial indicators for nature conservation from European to local scale. Ecological Indicators 5: 322-338.

58. Bodesmo M, Pacicco L, Romano B, Ranfa A (2012) The role of environmental and socio-demographic indicators in the analysis of land use changes in a protected area of the Natura 2000 Network: the case study of Lake Trasimeno, Umbria, Central Italy. Environmental Monitoring and Assessment 184: 831-843.

59. Boitani L, Falcucci A, Maiorano L, Rondinini C (2007) Ecological networks as conceptual frameworks or operational tools in conservation. Conservation Biology 21: 1414-1422.

60. Bonanno G (2013) Adaptive management as a tool to improve the conservation of endemic floras: the case of Sicily, Malta and their satellite islands. Biodiversity and Conservation 22: 1317-1354.

61. Bonanomi G, Incerti G, Allegrezza M (2013) Assessing the impact of land abandonment, nitrogen enrichment and fairy-ring fungi on plant diversity of Mediterranean grasslands. Biodiversity and Conservation 22: 2285-2304.

62. Bonte D, Criel P, Vanhoutte L, Van Thournout I, Maelfait JP (2004) The importance of habitat productivity, stability and heterogeneity for spider species richness in coastal grey dunes along the North Sea and its implications for conservation. Biodiversity and Conservation 13: 2119-2134.

63. Borges PAV, Aguiar C, Amaral J, Amorim IR, Andre G, et al. (2005) Ranking protected areas in the Azores using standardised sampling of soil epigean arthropods. Biodiversity and Conservation 14: 2029-2060.

64. Borgstrom S (2012) Legitimacy Issues in Finnish Wolf Conservation. Journal of Environmental Law 24: 451-476.

65. Borzel T, Buzogany A (2010) Environmental organisations and the Europeanisation of public policy in Central and Eastern Europe: the case of biodiversity governance. Environmental Politics 19: 708-735.

66. Bosso L, Rebelo H, Garonna AP, Russo D (2013) Modelling geographic distribution and detecting conservation gaps in Italy for the threatened beetle Rosalia alpina. Journal for Nature Conservation 21: 72-80.

67. Bouchet P, Falkner G, Seddon MB (1999) Lists of protected land and freshwater molluscs in the Bern Convention and European Habitats Directive: are they relevant to conservation? Biological Conservation 90: 21-31.

68. Bragazza L (2009) Conservation priority of Italian Alpine habitats: a floristic approach based on potential distribution of vascular plant species. Biodiversity and Conservation 18: 2823-2835.

69. Branquart E, Verheyen K, Latham J (2008) Selection criteria of protected forest areas in Europe: The theory and the real world. Biological Conservation 141: 2795-2806.

70. Briggs BDJ, Hill DA, Gosler AG (2012) Habitat selection and waterbody-complex use by wintering Gadwall and Shoveler in South West London: Implications for the designation and management of multi-site protected areas. Journal for Nature Conservation 20: 200-210.

71. Brown AE (2000) Conservation objectives and targets for freshwater sites. Journal of the Chartered Institution of Water and Environmental Management 14: 240-245.

72. Brullo C, Minissale P, Sciandrello S, Spampinato G (2011) Phytogeographic survey on the endemic vascular flora of the Hyblaean territory (SE Sicily, Italy). Acta Botanica Gallica 158: 617-631.

73. Brusa G, Cerabolini BEL (2009) Ecological factors affecting plant species and travertine deposition in petrifying springs from an Italian 'Natura 2000' site. Botanica Helvetica 119: 113-123.

74. Brusconi S, Bertocchi S, Renai B, Scalici M, Souty-Grosset C, et al. (2008) Conserving indigenous crayfish: stock assessment and habitat requirements in the threatened Austropotamobius italicus. Aquatic Conservation-Marine and Freshwater Ecosystems 18: 1227-1239.

75. Bryan S (2012) Contested boundaries, contested places: The Natura 2000 network in Ireland. Journal of Rural Studies 28: 80-94.

76. Buck O, Klink A, Millan VG, Pakzad K, Muterthies A (2013) Image Analysis Methods to Monitor Natura 2000 Habitats at Regional Scales - the MS.MONINA State Service Example in Schleswig-Holstein, Germany. Photogrammetrie Fernerkundung Geoinformation: 415-426.

77. Buffa G, Villani M (2012) Are the ancient forests of the Eastern Po Plain large enough for a long term conservation of herbaceous nemoral species? Plant Biosystems 146: 970-984.

78. Burmeier S, Jensen K (2008) Is the endangered Apium repens (Jacq.) Lag. rare because of a narrow regeneration niche? Plant Species Biology 23: 111-118.

79. Burrascano S, Anzellotti I, Carli E, Del Vico E, Facioni L, et al. (2013) Drivers of beta-diversity variation in Bromus erectus semi-natural dry grasslands. Applied Vegetation Science 16: 404-416.

80. Buse J, Ranius T, Assmann T (2008) An endangered longhorn beetle associated with old oaks and its possible role as an ecosystem engineer. Conservation Biology 22: 329-337.

81. Buse J, Schroder B, Assmann T (2007) Modelling habitat and spatial distribution of an endangered longhorn beetle - A case study for saproxylic insect conservation. Biological Conservation 137: 372-381.

82. Butler JRA, Middlemas SJ, McKelvey SA, McMyn I, Leyshon B, et al. (2008) The Moray Firth Seal Management Plan: an adaptive framework for balancing the conservation of seals, salmon, fisheries and wildlife tourism in the UK. Aquatic Conservation-Marine and Freshwater Ecosystems 18: 1025-1038.

83. Cacabelos E, Dominguez M, Troncoso JS (2009) Trophic structure of soft-bottom macrobenthos in an inlet in north-western Spain. Journal of the Marine Biological Association of the United Kingdom 89: 439-447.

84. Caci G, Biscaccianti AB, Cistrone L, Bosso L, Garonna AP, et al. (2013) Spotting the right spot: computer-aided individual identification of the threatened cerambycid beetle Rosalia alpina. Journal of Insect Conservation 17: 787-795.

85. Cai M, Pettenella D (2013) Protecting biodiversity outside protected areas: Can agricultural landscapes contribute to bird conservation on Natura 2000 in Italy? Journal of Environmental Engineering and Landscape Management 21: 1-11.

86. Campanaro A, Toni I, Hardersen S, Grasso DA (2011) Monitoring of Lucanus cervus by means of Remains of Predation (Coleoptera: Lucanidae). Entomologia Generalis 33: 79-89.

87. Canadas A, Sagarminaga R, De Stephanis R, Urquiola E, Hammond PS (2005) Habitat preference modelling as a conservation took proposals for marine protected areas for cetaceans in southern Spanish waters. Aquatic Conservation-Marine and Freshwater Ecosystems 15: 495-521.

88. Canadas A, Sagarminaga R, Garcia-Tiscar S (2002) Cetacean distribution related with depth and slope in the Mediterranean waters off southern Spain. Deep-Sea Research Part I-Oceanographic Research Papers 49: 2053-2073.

89. Cano L, Campos JA, Garcia-Magro D, Herrera M (2013) Replacement of estuarine communities by an exotic shrub: distribution and invasion history of Baccharis halimifolia in Europe. Biological Invasions 15: 1183-1188.

90. Cantarello E, Newton A (2008) Towards cost-effective indicators to maintain Natura 2000 sites in favourable conservation status. Preliminary results from Cansiglio and New Forest. Iforest-Biogeosciences and Forestry 1: 75-80.

91. Cantarello E, Newton AC (2008) Identifying cost-effective indicators to assess the conservation status of forested habitats in Natura 2000 sites. Forest Ecology and Management 256: 815-826.

92. Carbone F (2012) Forestry indemnity: a regional case study. European Journal of Forest Research 131: 119-129.

93. Cardoso P (2012) Habitats Directive species lists: urgent need of revision. Insect Conservation and Diversity 5: 169-174.

94. Carpaneto GM, Mazziotta A, Coletti G, Luiselli L, Audisio P (2010) Conflict between insect conservation and public safety: the case study of a saproxylic beetle (Osmoderma eremita) in urban parks. Journal of Insect Conservation 14: 555-565.

95. Carranza ML, Acosta ATR, Stanisci A, Pirone G, Ciaschetti G (2008) Ecosystem classification for EU habitat distribution assessment in sandy coastal environments: An application in central Italy. Environmental Monitoring and Assessment 140: 99-107.

96. Carranza ML, Frate L, Paura B (2012) Structure, ecology and plant richness patterns in fragmented beech forests. Plant Ecology & Diversity 5: 541-551.

97. Carreno MF, Esteve MA, Martinez J, Palazon JA, Pardo MT (2008) Habitat changes in coastal wetlands associated to hydrological changes in the watershed. Estuarine Coastal and Shelf Science 77: 475-483.

98. Carss D, Spears BM, Quinn L, Cooper R (2012) Long-term variations in waterfowl populations in Loch Leven: identifying discontinuities between local and national trends. Hydrobiologia 681: 85-104.

99. Castro P (2012) Legal Innovation for Social Change: Exploring Change and Resistance to Different Types of Sustainability Laws. Political Psychology 33: 105-121.

100. Castro P, Mouro C (2011) Psycho-Social Processes in Dealing with Legal Innovation in the Community: Insights from Biodiversity Conservation. American Journal of Community Psychology 47: 362-373.

101. Castro P, Mouro C, Gouveia R (2012) The Conservation of Biodiversity in Protected Areas: Comparing the Presentation of Legal Innovations in the National and the Regional Press. Society & Natural Resources 25: 539-555.

102. Cent J, Mertens C, Niedzialkowski K (2013) Roles and impacts of non-governmental organizations in Natura 2000 implementation in Hungary and Poland. Environmental Conservation 40: 119-128.

103. Cereghino R, Biggs J, Oertli B, Declerck S (2008) The ecology of European ponds: defining the characteristics of a neglected freshwater habitat. Hydrobiologia 597: 1-6.

104. Chan JCW, Beckers P, Spanhove T, Vanden Borre J (2012) An evaluation of ensemble classifiers for mapping Natura 2000 heathland in Belgium using spaceborne angular hyperspectral (CHRIS/Proba) imagery. International Journal of Applied Earth Observation and Geoinformation 18: 13-22.

105. Chefaoui RM, Hortal J, Lobo JM (2005) Potential distribution modelling, niche characterization and conservation status assessment using GIS tools: a case study of Iberian Copris species. Biological Conservation 122: 327-338.

106. Chefaoui RM, Lobo JM (2007) Assessing the conservation status of an Iberian moth using pseudo-absences. Journal of Wildlife Management 71: 2507-2516.

107. Chiarucci A, Bacaro G, Filibeck G, Landi S, Maccherini S, et al. (2012) Scale dependence of plant species richness in a network of protected areas. Biodiversity and Conservation 21: 503-516.

108. Chiarucci A, Bacaro G, Rocchini D (2008) Quantifying plant species diversity in a Natura 2000 network: Old ideas and new proposals. Biological Conservation 141: 2608-2618.

109. Chiarucci A, Bacaro G, Vannini A, Rocchini D (2008) Quantifying species richness at multiple spatial scales in a Natura 2000 network. Community Ecology 9: 185-192.

110. Chisamera G, Buzan EV, Sahlean T, Murariu D, Zupan S, et al. (2014) Bukovina blind mole rat Spalax graecus revisited: phylogenetics, morphology, taxonomy, habitat associations and conservation. Mammal Review 44: 19-29.

111. Christensen P, Kornov L (2011) EIA screening and nature protection in Denmark. Journal of Environmental Management 92: 1097-1103.

112. Cianfrani C, Le Lay G, Maiorano L, Satizabal HF, Loy A, et al. (2011) Adapting global conservation strategies to climate change at the European scale: The otter as a flagship species. Biological Conservation 144: 2068-2080.

113. Ciccarelli D, Bacaro G, Chiarucci A (2012) Coastline Dune Vegetation Dynamics: Evidence of No Stability. Folia Geobotanica 47: 263-275.

114. Cogalniceanu D, Cogalniceanu GC (2010) An enlarged European Union challenges priority settings in conservation. Biodiversity and Conservation 19: 1471-1483.

115. Cogoni A, Scrugli A, Cortis P (2009) Bryophyte flora of some temporary pools in Sardinia and Corsica. Plant Biosystems 143: S97-S103.

116. Corell H, Moksnes PO, Engqvist A, Doos K, Jonsson PR (2012) Depth distribution of larvae critically affects their dispersal and the efficiency of marine protected areas. Marine Ecology Progress Series 467: 29-+.

117. Cowx IG, Harvey JP, Noble RA, Nunn AD (2009) Establishing survey and monitoring protocols for the assessment of conservation status of fish populations in river Special Areas of Conservation in the UK. Aquatic Conservation-Marine and Freshwater Ecosystems 19: 96-103.

118. Cronin M, Duck C, Cadhla OO, Nairn R, Strong D, et al. (2007) An assessment of population size and distribution of harbour seals in the Republic of Ireland during the moult season in August 2003. Journal of Zoology 273: 131-139.

119. Cronin MA, Duck CD, Cadhla OO (2007) Aerial surveying of grey seat breeding colonies on the blasket islands, Co. kerry, the inishkea group, Co. Mayo and the Donegal coast, Ireland. Journal for Nature Conservation 15: 73-83.

120. Cruz A, Benedicto J, Gil A (2011) Socio-economic Benefits of Natura 2000 in Azores Islands - a Case Study approach on ecosystem services provided by a Special Protected Area. Journal of Coastal Research: 1955-1959.

121. Culmsee H, Schmidt M, Schmiedel I, Schacherer A, Meyer P, et al. (2014) Predicting the distribution of forest habitat types using indicator species to facilitate systematic conservation planning. Ecological Indicators 37: 131-144.

122. D'Amen M, Bombi P, Campanaro A, Zapponi L, Bologna MA, et al. (2013) Protected areas and insect conservation: Questioning the effectiveness of Natura 2000 network for saproxylic beetles in Italy. Animal Conservation 16: 370-378.

123. D'Amen M, Bombi P, Pearman PB, Schmatz DR, Zimmermann NE, et al. (2011) Will climate change reduce the efficacy of protected areas for amphibian conservation in Italy? Biological Conservation 144: 989-997.

124. de Bello F, Lavorel S, Gerhold P, Reier U, Partel M (2010) A biodiversity monitoring framework for practical conservation of grasslands and shrublands. Biological Conservation 143: 9-17.

125. de la Montana E, Benayas JMR, Vasques A, Razola I, Cayuela L (2011) Conservation planning of vertebrate diversity in a Mediterranean agricultural-dominant landscape. Biological Conservation 144: 2468-2478.

126. De Luca E, Novelli C, Barbato F, Menegoni P, Iannetta M, et al. (2011) Coastal dune systems and disturbance factors: monitoring and analysis in central Italy. Environmental Monitoring and Assessment 183: 437-450.

127. De Nooij RJW, Lotterman KM, de Sande P, Pelsma T, Leuven R, et al. (2006) Validity and sensitivity of a model for assessment of impacts of river floodplain reconstruction on protected and endangered species. Environmental Impact Assessment Review 26: 677-695.

128. De Santo EM, Jones PJS (2007) Offshore marine conservation policies in the North East Atlantic: Emerging tensions and opportunities. Marine Policy 31: 336-347.

129. del Barrio G, Puigdefabregas J, Sanjuan ME, Stellmes M, Ruiz A (2010) Assessment and monitoring of land condition in the Iberian Peninsula, 1989-2000. Remote Sensing of Environment 114: 1817-1832.

130. Delalieux S, Somers B, Haest B, Spanhove T, Vanden Borre J, et al. (2012) Heathland conservation status mapping through integration of hyperspectral mixture analysis and decision tree classifiers. Remote Sensing of Environment 126: 222-231.

131. Delgado MP, Morales MB, Traba J, De la Morena ELG (2009) Determining the effects of habitat management and climate on the population trends of a declining steppe bird. Ibis 151: 440-451.

132. Della Bella V, Bazzanti M, Chiarotti F (2005) Macroinvertebrate diversity and conservation status of Mediterranean ponds in Italy: water permanence and mesohabitat influence. Aquatic Conservation-Marine and Freshwater Ecosystems 15: 583-600.

133. Dennis RLH, Dapporto L, Shreeve TG, John E, Coutsis JG, et al. (2008) Butterflies of European islands: the implications of the geography and ecology of rarity and endemicity for conservation. Journal of Insect Conservation 12: 205-236.

134. Denoel M (2012) Newt decline in Western Europe: highlights from relative distribution changes within guilds. Biodiversity and Conservation 21: 2887-2898.

135. Denys L, Van Wichelen J, Packet J, Louette G (2014) Implementing ecological potential of lakes for the Water Framework Directive-Approach in Flanders (northern Belgium). Limnologica 45: 38-49.

136. Devictor V, Godet L, Julliard R, Couvet D, Jiguet F (2007) Can common species benefit from protected areas? Biological Conservation 139: 29-36.

137. Dias E, Elias RB, Nunes V (2004) Vegetation mapping and nature conservation: a case study in Terceira Island (Azores). Biodiversity and Conservation 13: 1519-1539.

138. Dias E, Melo C (2010) Factors influencing the distribution of Azorean mountain vegetation: implications for nature conservation. Biodiversity and Conservation 19: 3311-3326.

139. Diaz-Gomez DL, Toxopeus AG, Groen TA, Munoz AR, Skidmore AK, et al. (2013) Measuring the Insecurity Index of species in networks of protected areas using species distribution modeling and fuzzy logic: The case of raptors in Andalusia. Ecological Indicators 26: 174-182.

140. Diaz-Varela RA, Alvarez-Alvarez P, Diaz-Varela E, Calvo-Iglesias S (2011) Prediction of stand quality characteristics in sweet chestnut forests in NW Spain by combining terrain attributes, spectral textural features and landscape metrics. Forest Ecology and Management 261: 1962-1972.

141. Diesing M, Coggan R, Vanstaen K (2009) Widespread rocky reef occurrence in the central English Channel and the implications for predictive habitat mapping. Estuarine Coastal and Shelf Science 83: 647-658.

142. Dimitrakopoulos PG, Jones N, Iosifides T, Florokapi I, Lasda O, et al. (2010) Local attitudes on protected areas: Evidence from three Natura 2000 wetland sites in Greece. Journal of Environmental Management 91: 1847-1854.

143. Dimitrakopoulos PG, Memtsas D, Troumbis AY (2004) Questioning the effectiveness of the Natura 2000 Special Areas of Conservation strategy: the case of Crete. Global Ecology and Biogeography 13: 199-207.

144. Dimitriou E, Karaouzas I, Skoulikidis N, Zacharias I (2006) Assessing the environmental status of Mediterranean temporary ponds in Greece. Annales De Limnologie-International Journal of Limnology 42: 33-41.

145. Dimitriou E, Zacharias I (2010) Identifying microclimatic, hydrologic and land use impacts on a protected wetland area by using statistical models and GIS techniques. Mathematical and Computer Modelling 51: 200-205.

146. Dostalek T, Munzbergova Z, Plackova I (2010) Genetic diversity and its effect on fitness in an endangered plant species, Dracocephalum austriacum L. Conservation Genetics 11: 773-783.

147. Drakou EG, Kallimanis AS, Mazaris AD, Apostolopoulou E, Pantis JD (2011) Habitat type richness associations with environmental variables: a case study in the Greek Natura 2000 aquatic ecosystems. Biodiversity and Conservation 20: 929-943.

148. Drechsler M, Johst K, Ohl C, Watzold F (2007) Designing cost-effective payments for conservation measures to generate spatiotemporal habitat heterogeneity. Conservation Biology 21: 1475-1486.

149. Drechsler M, Watzold F, Johst K, Bergmann H, Settele J (2007) A model-based approach for designing cost-effective compensation payments for conservation of endangered species in real landscapes. Biological Conservation 140: 174-186.

150. Ducasse JJ, Brustel H (2008) Saproxylic beetles in the Gresigne forest management. Revue D Ecologie-La Terre Et La Vie: 75-80.

151. Durell S, McGrorty S, West AD, Clarke RT, Goss-Custard JD, et al. (2005) A strategy for baseline monitoring of estuary Special Protection Areas. Biological Conservation 121: 289-301.

152. Early R, Thomas CD (2007) Multispecies conservation planning: identifying landscapes for the conservation of viable populations using local and continental species priorities. Journal of Applied Ecology 44: 253-262.

153. Ecke F, Hellsten S, Mjelde M, Kuoppala M, Schlacke S (2010) Potential conflicts between environmental legislation and conservation exemplified by aquatic macrophytes. Hydrobiologia 656: 107-115.

154. Edman T, Angelstam P, Mikusinski G, Roberge JM, Sikora A (2011) Spatial planning for biodiversity conservation: Assessment of forest landscapes' conservation value using umbrella species requirements in Poland. Landscape and Urban Planning 102: 16-23.

155. Edwards D, Jensen FS, Marzano M, Mason B, Pizzirani S, et al. (2011) A theoretical framework to assess the impacts of forest management on the recreational value of European forests. Ecological Indicators 11: 81-89.

156. Ejrnaes R, Bruun HH, Aude E, Buchwald E (2004) Developing a classifier for the habitats directive grassland types in Denmark using species lists for prediction. Applied Vegetation Science 7: 71-80.

157. Ekebom J, Erkkila A (2003) Using aerial photography for identification of marine and coastal habitats under the EU's Habitats Directive. Aquatic Conservation-Marine and Freshwater Ecosystems 13: 287-304.

158. Embling CB, Gillibrand PA, Gordon J, Shrimpton J, Stevick PT, et al. (2010) Using habitat models to identify suitable sites for marine protected areas for harbour porpoises (Phocoena phocoena). Biological Conservation 143: 267-279.

159. Eppink FV, Watzold F (2009) Shedding light on the hidden costs of the Habitats Directive: the case of hamster conservation in Germany. Biodiversity and Conservation 18: 795-810.

160. Ernandes P, Marchiori S (2013) Mediterranean temporary ponds in Puglia (South Italy): a "joyau floristique" to protect. Acta Botanica Gallica 160: 53-64.

161. Evans D (2010) Interpreting the habitats of Annex I: past, present and future. Acta Botanica Gallica 157: 677-686.

162. Evans D, Demeter A, Gajdos P, Halada L (2013) Adapting environmental conservation legislation for an enlarged European Union: experience from the Habitats Directive. Environmental Conservation 40: 97-107.

163. Faria N, Rabaca JE, Morales MB (2012) The importance of grazing regime in the provision of breeding habitat for grassland birds: The case of the endangered little bustard (Tetrax tetrax). Journal for Nature Conservation 20: 211-218.

164. Faria N, Rabaqa JE (2004) Breeding habitat modelling of the Little Bustard Tetrax tetrax in the site of community importance of Cabrela (Portugal). Ardeola 51: 331-343.

165. Farris E, Fenu G, Baccheta G (2012) Mediterranean Taxus baccata woodlands in Sardinia: a characterization of the EU priority habitat 9580. Phytocoenologia 41: 231-246.

166. Farris E, Filigheddu R (2008) Effects of browsing in relation to vegetation cover on common yew (Taxus baccata L.) recruitment in Mediterranean environments. Plant Ecology 199: 309-318.

167. Farris E, Filigheddu R, Deiana P, Farris GA, Garau G (2010) Short-term effects on sheep pastureland due to grazing abandonment in a Western Mediterranean island ecosystem: A multidisciplinary approach. Journal for Nature Conservation 18: 258-267.

168. Farris E, Pisanu S, Ceccherelli G, Filigheddu R (2009) Effects of the management regime on the performance of the endangered Mediterranean Centaurea horrida Badaro (Asteraceae). Journal for Nature Conservation 17: 15-24.

169. Farris E, Pisanu S, Ceccherelli G, Filigheddu R (2013) Human trampling effects on Mediterranean coastal dune plants. Plant Biosystems 147: 1043-1051.

170. Farris E, Secchi Z, Rosati L, Filigheddu R (2013) Are all pastures eligible for conservation? A phytosociological survey of the Sardinian-Corsican Province as a basic tool for the Habitats Directive. Plant Biosystems 147: 931-946.

171. Fattorini S, Santoro R, Maurizi E, Acosta ATR, Di Giulio A (2012) Environmental tuning of an insect ensemble: The tenebrionid beetles inhabiting a Mediterranean coastal dune zonation. Comptes Rendus Biologies 335: 708-711.

172. Fehervari P, Solt S, Palatitz P, Barna K, Agoston A, et al. (2012) Allocating active conservation measures using species distribution models: a case study of red-footed falcon breeding site management in the Carpathian Basin. Animal Conservation 15: 648-657.

173. Feola S, Carranza ML, Schaminee JHJ, Janssen JAM, Acosta ATR (2011) EU habitats of interest: an insight into Atlantic and Mediterranean beach and foredunes. Biodiversity and Conservation 20: 1457-1468.

174. Ferranti F, Beunen R, Speranza M (2010) Natura 2000 Network: A Comparison of the Italian and Dutch Implementation Experiences. Journal of Environmental Policy & Planning 12: 293-314.

175. Ferreira AF, Quintella BR, Maia C, Mateus CS, Alexandre CM, et al. (2013) Influence of macrohabitat preferences on the distribution of European brook and river lampreys: Implications for conservation and management. Biological Conservation 159: 175-186.

176. Feys S, Guelinckx R, Verdonckt F, Louette G (2013) Successful reproduction of Hen Harrier Circus cyaneus in intensive arable farmland (central-east Belgium). Belgian Journal of Zoology 143: 142-147.

177. Fischer HS, Winter S, Lohberger E, Jehl H, Fischer A (2013) Improving Transboundary Maps of Potential Natural Vegetation Using Statistical Modeling Based on Environmental Predictors. Folia Geobotanica 48: 115-135.

178. Fliervoet JM, Van den Born RJG, Smits AJM, Knippenberg L (2013) Combining safety and nature: A multi-stakeholder perspective on integrated floodplain management. Journal of Environmental Management 128: 1033-1042.

179. Fock HO (2011) Natura 2000 and the European Common Fisheries Policy. Marine Policy 35: 181-188.

180. Fock HO, Kloppmann M, Stelzenmuller V (2011) Linking marine fisheries to environmental objectives: a case study on seafloor integrity under European maritime policies. Environmental Science & Policy 14: 289-300.

181. Forster M, Frick A, Walentowski H, Kleinschmit B (2008) Approaches to utilising QuickBird data for the monitoring of NATURA 2000 habitats. Community Ecology 9: 155-168.

182. Franco A, Franzoi P, Malavasi S, Zucchetta M, Torricelli P (2012) Population and habitat status of two endemic sand gobies in lagoon marshes - Implications for conservation. Estuarine Coastal and Shelf Science 114: 31-40.

183. Frandsen EL, Jensen NH, Veihe A, Frederiksen P (2007) Young dry grassland ecosystems in Denmark: development in soil nutrient pools and root characteristics. Geografisk Tidsskrift-Danish Journal of Geography 107: 17-28.

184. Frank G, Muller F (2003) Voluntary approaches in protection of forests in Austria. Environmental Science & Policy 6: 261-269.

185. Franke J, Keuck V, Siegert F (2012) Assessment of grassland use intensity by remote sensing to support conservation schemes. Journal for Nature Conservation 20: 125-134.

186. Fraschetti S, Terlizzi A, Boero F (2008) How many habitats are there in the sea (and where)? Journal of Experimental Marine Biology and Ecology 366: 109-115.

187. Funk A, Gschopf C, Blaschke AP, Weigelhofer G, Reckendorfer W (2013) Ecological niche models for the evaluation of management options in an urban floodplain-conservation vs. restoration purposes. Environmental Science & Policy 34: 79-91.

188. Galantinho A, Mira A (2009) The influence of human, livestock, and ecological features on the occurrence of genet (Genetta genetta): a case study on Mediterranean farmland. Ecological Research 24: 671-685.

189. Ganatsas P, Thanasis G (2010) Pinus halepensis invasion in Pinus pinea habitat in Strofylia forest (Site of NATURA 2000 network), southern Greece. Journal for Nature Conservation 18: 106-117.

190. Ganatsas P, Tsakaldimi M, Katsaros D (2013) Natural resource management in national parks: a management assessment of a Natura 2000 wetlands site in Kotychi-Strofylia, southern Greece. International Journal of Sustainable Development and World Ecology 20: 152-165.

191. Ganatsas P, Tsakaldimi M, Thanos C (2008) Seed and cone diversity and seed germination of Pinus pinea in Strofylia Site of the Natura 2000 Network. Biodiversity and Conservation 17: 2427-2439.

192. Garcia MB, Guzman D, Goni D (2002) An evaluation of the status of five threatened plant species in the Pyrenees. Biological Conservation 103: 151-161.

193. Garrido J, Munilla I (2008) Aquatic Coleoptera and Hemiptera assemblages in three coastal lagoons of the NW Iberian Peninsula: assessment of conservation value and response to environmental factors. Aquatic Conservation-Marine and Freshwater Ecosystems 18: 557-569.

194. Garthe S, Markones N, Mendel B, Sonntag N, Krause JC (2012) Protected areas for seabirds in German offshore waters: Designation, retrospective consideration and current perspectives. Biological Conservation 156: 126-135.

195. Gaston KJ, Jackson SE, Nagy A, Cantu-Salazar L, Johnson M (2008) Protected areas in Europe - Principle and practice. Year in Ecology and Conservation Biology 2008 1134: 97-119.

196. Gauthier P, Foulon Y, Jupille O, Thompson JD (2013) Quantifying habitat vulnerability to assess species priorities for conservation management. Biological Conservation 158: 321-325.

197. Gegout JC, Coudun C (2012) The right releve in the right vegetation unit: a new typicality index to reproduce expert judgement with an automatic classification programme. Journal of Vegetation Science 23: 24-32.

198. Giakoumi S, Grantham HS, Kokkoris GD, Possingham HP (2011) Designing a network of marine reserves in the Mediterranean Sea with limited socio-economic data. Biological Conservation 144: 753-763.

199. Giakoumi S, Katsanevakis S, Vassilopoulou V, Panayotidis P, Kavadas S, et al. (2012) Could European marine conservation policy benefit from systematic conservation planning? Aquatic Conservation-Marine and Freshwater Ecosystems 22: 762-775.

200. Gil A, Calado H, Costa LT, Bentz J, Fonseca C, et al. (2011) A Methodological Proposal for the Development of Natura 2000 Sites Management Plans. Journal of Coastal Research: 1326-1330.

201. Godet L, Devictor V, Jiguet F (2007) Estimating relative population size included within protected areas. Biodiversity and Conservation 16: 2587-2598.

202. Gonzalez-Estebanez FJ, Garcia-Tejero S, Mateo-Tomas P, Olea PP (2011) Effects of irrigation and landscape heterogeneity on butterfly diversity in Mediterranean farmlands. Agriculture Ecosystems & Environment 144: 262-270.

203. Gonzalvo J, Moutopoulos DK, Bearzi G, Stergiou KI (2011) Fisheries mismanagement in a Natura 2000 area in western Greece. Fisheries Management and Ecology 18: 25-38.

204. Gouix N, Brustel H (2012) Emergence trap, a new method to survey Limoniscus violaceus (Coleoptera: Elateridae) from hollow trees. Biodiversity and Conservation 21: 421-436.

205. Gouix N, Mertlik J, Jarzabek-Muller A, Nemeth T, Brustel H (2012) Known status of the endangered western Palaearctic violet click beetle (Limoniscus violaceus) (Coleoptera). Journal of Natural History 46: 769-802.

206. Grall J, Hall-Spencer JM (2003) Problems facing maerl conservation in Brittany. Aquatic Conservation-Marine and Freshwater Ecosystems 13: S55-S64.

207. Grammatikopoulou I, Olsen SB (2013) Accounting protesting and warm glow bidding in Contingent Valuation surveys considering the management of environmental goods - An empirical case study assessing the value of protecting a Natura 2000 wetland area in Greece. Journal of Environmental Management 130: 232-241.

208. Grashof-Bokdam CJ, Chardon JP, Vos CC, Foppen RPB, WallisDeVries M, et al. (2009) The synergistic effect of combining woodlands and green veining for biodiversity. Landscape Ecology 24: 1105-1121.

209. Grava T, Mathevon N, Place E, Balluet P (2008) Individual acoustic monitoring of the European Eagle Owl Bubo bubo. Ibis 150: 279-287.

210. Graziano R, Gilberto P, Alessandro F (2009) A rapid and cost-effective tool for managing habitats of the European Natura 2000 network: a case study in the Italian Alps. Biodiversity and Conservation 18: 1375-1388.

211. Green DS, Crowe TP (2013) Physical and biological effects of introduced oysters on biodiversity in an intertidal boulder field. Marine Ecology Progress Series 482: 119-132.

212. Grigoriadis N, Panagopoulos A, Meliadis I, Spyroglou G, Stathaki S (2009) Habitat and hydrological-hydrochemical characteristics of the Agras wetland (Northern Greece). Plant Biosystems 143: 162-172.

213. Grigoriadis N, Petermann J, Schroder E, Spyroglou G (2012) Contribution to the assessment of the conservation status of spruce forests in Greece. Journal of Biological Research-Thessaloniki 17: 57-67.

214. Grodzinska-Jurczak M, Cent J (2011) Expansion of Nature Conservation Areas: Problems with Natura 2000 Implementation in Poland? Environmental Management 47: 11-27.

215. Guinda X, Juanes JA, Puente A, Echavarri-Erasun B (2012) Spatial distribution pattern analysis of subtidal macroalgae assemblages by a non-destructive rapid assessment method. Journal of Sea Research 67: 34-43.

216. Gurrutxaga M, Lozano PJ, del Barrio G (2010) GIS-based approach for incorporating the connectivity of ecological networks into regional planning. Journal for Nature Conservation 18: 318-326.

217. Gurrutxaga M, Rubio L, Saura S (2011) Key connectors in protected forest area networks and the impact of highways: A transnational case study from the Cantabrian Range to the Western Alps (SW Europe). Landscape and Urban Planning 101: 310-320.

218. Guthlin D, Knauer F, Kneib T, Kuchenhoff H, Kaczensky P, et al. (2011) Estimating habitat suitability and potential population size for brown bears in the Eastern Alps. Biological Conservation 144: 1733-1741.

219. Gutierrez D, Menendez R (2007) Regional hotspots of butterfly diversity in a protected area: Are they indicators of unique assemblages and areas with more species of conservation concern? Acta Oecologica-International Journal of Ecology 32: 301-311.

220. Hajek M, Hajkova P, Apostolova I, Horsak M, Rozbrojova Z, et al. (2010) The insecure future of Bulgarian refugial mires: economic progress versus Natura 2000. Oryx 44: 539-546.

221. Halada L, Evans D, Romao C, Petersen JE (2011) Which habitats of European importance depend on agricultural practices? Biodiversity and Conservation 20: 2365-2378.

222. Hallsworth S, Dore AJ, Bealey WI, Dragosits U, Vieno M, et al. (2010) The role of indicator choice in quantifying the threat of atmospheric ammonia to the 'Natura 2000' network. Environmental Science & Policy 13: 671-687.

223. Hammond PS, Macleod K, Berggren P, Borchers DL, Burt L, et al. (2013) Cetacean abundance and distribution in European Atlantic shelf waters to inform conservation and management. Biological Conservation 164: 107-122.

224. Hardegen M, Bougault C, Quere E (2008) Cartography of habitats in Natura 2000 sites from Britain. Application to the maritime heathlands of Groix island and Crozon peninsula. Acta Botanica Gallica 155: 153-159.

225. Hartel T, Moga CI, David A, Coroiu I (2009) Species richness - pond area relationships of amphibians and birds in two Natura 2000 protected areas of Romania. Community Ecology 10: 159-164.

226. Hartel T, Nemes S, Cogalniceanu D, Ollerer K, Moga CI, et al. (2009) Pond and landscape determinants of Rana dalmatina population sizes in a Romanian rural landscape. Acta Oecologica-International Journal of Ecology 35: 53-59.

227. Haslett JR, Berry PM, Bela G, Jongman RHG, Pataki G, et al. (2010) Changing conservation strategies in Europe: a framework integrating ecosystem services and dynamics. Biodiversity and Conservation 19: 2963-2977.

228. Hernandez-Manrique OL, Numa C, Verdu JR, Galante E, Lobo JM (2012) Current protected sites do not allow the representation of endangered invertebrates: the Spanish case. Insect Conservation and Diversity 5: 414-421.

229. Hernando A, Tejera R, Velazquez J, Nunez MV (2010) Quantitatively defining the conservation status of Natura 2000 forest habitats and improving management options for enhancing biodiversity. Biodiversity and Conservation 19: 2221-2233.

230. Hiedanpaa J (2002) European-wide conservation versus local well-being: the reception of the Natura 2000 Reserve Network in Karvia, SW-Finland. Landscape and Urban Planning 61: 113-123.

231. Hiedanpaa J (2005) The edges of conflict and consensus: a case for creativity in regional forest policy in Southwest Finland. Ecological Economics 55: 485-498.

232. Hiedanpaa J, Bromley DW (2011) The Harmonization Game: Reasons and Rules in European Biodiversity Policy. Environmental Policy and Governance 21: 99-111.

233. Higgins RM, Vandeperre F, Perez-Ruzafa A, Santos RS (2008) Priorities for fisheries in marine protected area design and management: Implications for artisanal-type fisheries as found in southern Europe. Journal for Nature Conservation 16: 222-233.

234. Hirschnitz-Garbers M, Stoll-Kleemann S (2011) Opportunities and barriers in the implementation of protected area management: a qualitative meta-analysis of case studies from European protected areas. Geographical Journal 177: 321-334.

235. Hochkirch A, Schmitt T, Beninde J, Hiery M, Kinitz T, et al. (2013) How Much Biodiversity does Natura 2000 Cover? Conservation Letters 6: 470-471.

236. Hochkirch A, Schmitt T, Beninde J, Hiery M, Kinitz T, et al. (2013) Europe Needs a New Vision for a Natura 2020 Network. Conservation Letters 6: 462-467.

237. Holm TE, Laursen K (2009) Experimental disturbance by walkers affects behaviour and territory density of nesting Black-tailed Godwit Limosa limosa. Ibis 151: 77-87.

238. Horak J, Vavrova E, Chobot K (2010) Habitat preferences influencing populations, distribution and conservation of the endangered saproxylic beetle Cucujus cinnaberinus (Coleoptera: Cucujidae) at the landscape level. European Journal of Entomology 107: 81-88.

239. Horvath GF, Herczeg R (2013) Site occupancy response to natural and anthropogenic disturbances of root vole: Conservation problem of a vulnerable relict subspecies. Journal for Nature Conservation 21: 350-358.

240. Horvath Z, Ferenczi M, Mora A, Vad CF, Ambrus A, et al. (2012) Invertebrate food sources for waterbirds provided by the reconstructed wetland of Nyirkai-Hany, northwestern Hungary. Hydrobiologia 697: 59-72.

241. Hoyos D, Mariel P, Pascual U, Etxano I (2012) Valuing a Natura 2000 network site to inform land use options using a discrete choice experiment: An illustration from the Basque Country. Journal of Forest Economics 18: 329-344.

242. Hufkens K, Thoonen G, Vanden Borre J, Scheunders P, Ceulemans R (2010) Habitat reporting of a heathland site: Classification probabilities as additional information, a case study. Ecological Informatics 5: 248-255.

243. Hunter PD, Gilvear DJ, Tyler AN, Willby NJ, Kelly A (2010) Mapping macrophytic vegetation in shallow lakes using the Compact Airborne Spectrographic Imager (CASI). Aquatic Conservation-Marine and Freshwater Ecosystems 20: 717-727.

244. Immitzer M, Nopp-Mayr U, Zohmann M (2014) Effects of habitat quality and hiking trails on the occurrence of Black Grouse (Tetrao tetrix L.) at the northern fringe of alpine distribution in Austria. Journal of Ornithology 155: 173-181.

245. Ioja CI, Patroescu M, Rozylowicz L, Popescu VD, Verghelet M, et al. (2010) The efficacy of Romania's protected areas network in conserving biodiversity. Biological Conservation 143: 2468-2476.

246. Ioppolo G, Saija G, Salomone R (2013) From coastal management to environmental management: The sustainable eco-tourism program for the mid-western coast of Sardinia (Italy). Land Use Policy 31: 460-471.

247. Izco J, Amigo J, Pulgar I (2009) Violion caninae grasslands (Nardetea strictae) in the North and North-West of Spain. Acta Botanica Gallica 156: 437-454.

248. Jackson ALR (2011) Renewable energy vs. biodiversity: Policy conflicts and the future of nature conservation. Global Environmental Change-Human and Policy Dimensions 21: 1195-1208.

249. Jackson SF, Gaston KJ (2008) Incorporating private lands in conservation planning: Protected areas in Britain. Ecological Applications 18: 1050-1060.

250. Jackson SF, Kershaw M, Gaston KJ (2004) The performance of procedures for selecting conservation areas: waterbirds in the UK. Biological Conservation 118: 261-270.

251. Jaeschke A, Bittner T, Reineking B, Beierkuhnlein C (2013) Can they keep up with climate change? - Integrating specific dispersal abilities of protected Odonata in species distribution modelling. Insect Conservation and Diversity 6: 93-103.

252. Jantke K, Schleupner C, Schneider UA (2011) Gap analysis of European wetland species: priority regions for expanding the Natura 2000 network. Biodiversity and Conservation 20: 581-605.

253. Jimenez-Franco MV, Martinez JE, Pagan I, Calvo JF (2013) Factors determining territory fidelity in a migratory forest raptor, the Booted Eagle Hieraaetus pennatus. Journal of Ornithology 154: 311-318.

254. Jiricka A, Probstl U (2009) One common way - The strategic and methodological influence on environmental planning across Europe. Environmental Impact Assessment Review 29: 379-389.

255. Johnson DE, Bartlett J, Nash LA (2007) Coastal lagoon habitat re-creation potential in Hampshire, England. Marine Policy 31: 599-606.

256. Johnson MP, Crowe TP, McAllen R, Allcock AL (2008) Characterizing the marine Natura 2000 network for the Atlantic region. Aquatic Conservation-Marine and Freshwater Ecosystems 18: 86-97.

257. Jones PJS (1999) Marine nature reserves in Britain: past lessons, current status and future issues. Marine Policy 23: 375-396.

258. Jones-Walters L, Cil A (2011) Biodiversity and stakeholder participation. Journal for Nature Conservation 19: 327-329.

259. Jones-Walters L, Civic K (2013) European protected areas: Past, present and future. Journal for Nature Conservation 21: 122-124.

260. Junker M, Schmitt T (2010) Demography, dispersal and movement pattern of Euphydryas aurinia (Lepidoptera: Nymphalidae) at the Iberian Peninsula: an alarming example in an increasingly fragmented landscape? Journal of Insect Conservation 14: 237-246.

261. Jurc M, Ogris N, Pavlin R, Borkovic D (2008) Forest as a Habitat of Saproxylic Beetles on Natura 2000 Sites In Slovenia. Revue D Ecologie-La Terre Et La Vie: 61-74.

262. Juskaitis R (2008) Long-term common dormouse monitoring: effects of forest management on abundance. Biodiversity and Conservation 17: 3559-3565.

263. Juskiewicz-Swaczyna B, Choszcz D (2012) Effect of Habitat Quality on the Structure tf Populations of Pulsatilla patens (L.) Mill. (Ranunculaceae) - Rare And Endangered Species In European Flora. Polish Journal of Ecology 60: 567-576.

264. Kafyri A, Hovardas T, Poirazidis K (2012) Determinants of Visitor Pro-Environmental Intentions on Two Small Greek Islands: Is Ecotourism Possible at Coastal Protected Areas? Environmental Management 50: 64-76.

265. Kaiser MJ, Bergmann M, Hinz H, Galanidi M, Shucksmith R, et al. (2004) Demersal fish and epifauna associated with sandbank habitats. Estuarine Coastal and Shelf Science 60: 445-456.

266. Kallimanis AS, Mazaris AD, Tzanopoulos J, Halley JM, Pantis JD, et al. (2008) How does habitat diversity affect the species-area relationship? Global Ecology and Biogeography 17: 532-538.

267. Kallimanis AS, Ragia V, Sgardelis SP, Pantis JD (2007) Using regression trees to predict alpha diversity based upon geographical and habitat characteristics. Biodiversity and Conservation 16: 3863-3876.

268. Kallimanis AS, Tsiafouli MA, Pantis JD, Mazaris AD, Matsinos Y, et al. (2008) Arable land and habitat diversity in Natura 2000 sites in Greece. Journal of Biological Research-Thessaloniki 9: 55-66.

269. Kanongdate K, Schmidt M, Krawczynski R, Wiegleb G (2012) Has implementation of the precautionary principle failed to prevent biodiversity loss at the national level? Biodiversity and Conservation 21: 3307-3322.

270. Kati V, Dimopoulos P, Papaioannou H, Poirazidis K (2009) Ecological management of a Mediterranean mountainous reserve (Pindos National Park, Greece) using the bird community as an indicator. Journal for Nature Conservation 17: 47-59.

271. Kati V, Mani P, von Helversen O, Willemse F, Elsner N, et al. (2006) Human land use threatens endemic wetland species: the case of Chorthippus lacustris (La Greca and Messina 1975) (Orthoptera : Acrididae) in Epirus, Greece. Journal of Insect Conservation 10: 65-74.

272. Katsanevakis S, Poursanidis D, Issaris Y, Panou A, Petza D, et al. (2011) "Protected" marine shelled molluscs: thriving in Greek seafood restaurants. Mediterranean Marine Science 12: 429-438.

273. Keulartz J (2009) European Nature Conservation and Restoration Policy-Problems and Perspectives. Restoration Ecology 17: 446-450.

274. Kimmel K, Kull A, Salm JO, Mander U (2010) The status, conservation and sustainable use of Estonian wetlands. Wetlands Ecology and Management 18: 375-395.

275. Kindermann G, Gormally MJ (2013) Stakeholder perceptions of recreational and management impacts on protected coastal dune systems: A comparison of three European countries. Land Use Policy 31: 472-485.

276. Klar N, Fernandez N, Kramer-Schadt S, Herrmann M, Trinzen M, et al. (2008) Habitat selection models for European wildcat conservation. Biological Conservation 141: 308-319.

277. Klar N, Herrmann M, Kramer-Schadt S (2009) Effects and Mitigation of Road Impacts on Individual Movement Behavior of Wildcats. Journal of Wildlife Management 73: 631-638.

278. Klassert C, Mockel S (2013) Improving the Policy Mix: The Scope for Market-Based Instruments in EU Biodiversity Policy. Environmental Policy and Governance 23: 311-322.

279. Klauco M, Gregorova B, Stankov U, Markovic V, Lemenkova P (2013) Determination of ecological significance based on geostatistical assessment: a case study from the Slovak Natura 2000 protected area. Central European Journal of Geosciences 5: 28-42.

280. Kleinbauer I, Dullinger S, Peterseil J, Essl F (2010) Climate change might drive the invasive tree Robinia pseudacacia into nature reserves and endangered habitats. Biological Conservation 143: 382-390.

281. Knorn J, Kuemmerle T, Radeloff VC, Keeton WS, Gancz V, et al. (2013) Continued loss of temperate old-growth forests in the Romanian Carpathians despite an increasing protected area network. Environmental Conservation 40: 182-193.

282. Koljonen ML (2001) Conservation goals and fisheries management units for Atlantic salmon in the Baltic Sea area. Journal of Fish Biology 59: 269-288.

283. Konvicka M, Benes J, Cizek O, Kopecek F, Konvicka O, et al. (2008) How too much care kills species: Grassland reserves, agri-environmental schemes and extinction of Colias myrmidone (Lepidoptera : Pieridae) from its former stronghold. Journal of Insect Conservation 12: 519-525.

284. Kopec D, Michalska-Hejduk D, Krogulec E (2013) The relationship between vegetation and groundwater levels as an indicator of spontaneous wetland restoration. Ecological Engineering 57: 242-251.

285. Koutsos TM, Dimopoulos GC, Mamolos AP (2010) Spatial evaluation model for assessing and mapping impacts on threatened species in regions adjacent to Natura 2000 sites due to dam construction. Ecological Engineering 36: 1017-1027.

286. Kowalska A (2012) Changes In The Area Of Protected Plant Communities In The Middle Vistula River Valley In The Second Half Of The 20th Century. Polish Journal of Ecology 60: 19-29.

287. Kramer L (2009) The European Commission's Opinions underArticle 6(4) of the Habitats Directive. Journal of Environmental Law 21: 59-85.

288. Krause B, Culmsee H (2013) The significance of habitat continuity and current management on the compositional and functional diversity of grasslands in the uplands of Lower Saxony, Germany. Flora 208: 299-311.

289. Kros J, Frumau KFA, Hensen A, de Vries W (2011) Integrated analysis of the effects of agricultural management on nitrogen fluxes at landscape scale. Environmental Pollution 159: 3171-3182.

290. Kros J, Gies TJA, Voogd JCH, de Vries W (2013) Efficiency of agricultural measures to reduce nitrogen deposition in Natura 2000 sites. Environmental Science & Policy 32: 68-79.

291. Kuczynska A, Moorkens E (2010) Micro-hydrological and micro-meteorological controls on survival and population growth of the whorl snail Vertigo geyeri Lindholm, 1925 in groundwater fed wetlands. Biological Conservation 143: 1868-1875.

292. Kull T, Sammul M, Kull K, Lanno K, Tali K, et al. (2008) Necessity and reality of monitoring threatened European vascular plants. Biodiversity and Conservation 17: 3383-3402.

293. Kutnar L, Matijasic D, Pisek R (2011) Conservation status and potential threats to Natura 2000 forest habitats in Slovenia. Sumarski List 135: 215-231.

294. Laaksonen M, Murdoch K, Siitonen J, Varkonyi G (2010) Habitat associations of Agathidium pulchellum, an endangered old-growth forest beetle species living on slime moulds. Journal of Insect Conservation 14: 89-98.

295. Lacambra LCJ, de Una LF, Santos MM, de Lerma FBL, San Emeterio VE (2013) Structural characterization and analysis of the regeneration of woodlands dominated by Juniperus oxycedrus L. in west-central Spain. Plant Ecology 214: 61-73.

296. Lachat T, Wermelinger B, Gossner MM, Bussler H, Isacsson G, et al. (2012) Saproxylic beetles as indicator species for dead-wood amount and temperature in European beech forests. Ecological Indicators 23: 323-331.

297. Laffan B, O'Mahony J (2008) 'Bringing Politics Back In'. Domestic Conflict and the Negotiated Implementation of EU Nature Conservation Legislation in Ireland. Journal of Environmental Policy & Planning 10: 175-197.

298. Laikre L, Jansson M, Allendorf FW, Jakobsson S, Ryman N (2013) Hunting Effects on Favourable Conservation Status of Highly Inbred Swedish Wolves. Conservation Biology 27: 248-253.

299. Laine AM, Leppala M, Tarvainen O, Paatalo ML, Seppanen R, et al. (2011) Restoration of managed pine fens: effect on hydrology and vegetation. Applied Vegetation Science 14: 340-349.

300. Laiolo P, Tella JL (2006) Fate of unproductive and unattractive habitats: recent changes in Iberian steppes and their effects on endangered avifauna. Environmental Conservation 33: 223-232.

301. Langanke T, Burnett C, Lang S (2007) Assessing the mire conservation status of a raised bog site in Salzburg using object-based monitoring and structural analysis. Landscape and Urban Planning 79: 160-169.

302. Langston W, Chesman B, Burt G, Taylor M, Covey R, et al. (2006) Characterisation of the European Marine Sites in South West England: the Fal and Helford candidate Special Area of Conservation (cSAC). Hydrobiologia 555: 321-333.

303. Lazarina M, Sgardeli V, Kallimanis AS, Sgardelis SP (2013) An effort-based index of beta diversity. Methods in Ecology and Evolution 4: 217-225.

304. Ledoux L, Crooks S, Jordan A, Turner RK (2000) Implementing EU biodiversity policy: UK experiences. Land Use Policy 17: 257-268.

305. Lee M (2001) Coastal defence and the Habitats Directive: predictions of habitat change in England and Wales. Geographical Journal 167: 39-56.

306. Lengyel S, Deri E, Varga Z, Horvath R, Tothmeresz B, et al. (2008) Habitat monitoring in Europe: a description of current practices. Biodiversity and Conservation 17: 3327-3339.

307. Lengyel S, Kobler A, Kutnar L, Framstad E, Henry PY, et al. (2008) A review and a framework for the integration of biodiversity monitoring at the habitat level. Biodiversity and Conservation 17: 3341-3356.

308. Leone V, Lovreglio R (2004) Conservation of Mediterranean pine woodlands: scenarios and legislative tools. Plant Ecology 171: 221-235.

309. Li CZ, Kuuluvainen J, Pouta E, Rekola M, Tahvonen O (2004) Using choice experiments to value the natura 2000 nature conservation programs in Finland. Environmental & Resource Economics 29: 361-374.

310. Lik M (2010) The influence of habitat type on the population dynamics of ground beetles (Coleoptera: Carabidae) in marshland. Annales De La Societe Entomologique De France 46: 425-438.

311. Lison F, Palazon JA, Calvo JF (2013) Effectiveness of the Natura 2000 Network for the conservation of cave-dwelling bats in a Mediterranean region. Animal Conservation 16: 528-537.

312. Litskas VD, Karaolis CS, Menexes GC, Mamolos AP, Koutsos TM, et al. (2013) Variation of energy flow and greenhouse gas emissions in vineyards located in Natura 2000 sites. Ecological Indicators 27: 1-7.

313. Llusia D, Onate JJ (2005) Are the conservation requirements of pseudo-steppe birds adequately covered by Spanish agri-environmental schemes? An ex-ante assessment. Ardeola 52: 31-42.

314. Lombardi F, Klopcic M, Di Martino P, Tognetti R, Chirici G, et al. (2012) Comparison of forest stand structure and management of silver fir-European beech forests in the Central Apennines, Italy and in the Dinaric Mountains, Slovenia. Plant Biosystems 146: 114-123.

315. Lonergan M (2011) Potential biological removal and other currently used management rules for marine mammal populations: A comparison. Marine Policy 35: 584-589.

316. Lopez-Pantoja G, Dominguez L, Sanchez-Osorio I (2011) Analysis of Prinobius myardi Mulsant population dynamics in a Mediterranean cork oak stand. Annales De La Societe Entomologique De France 47: 260-268.

317. Louette G, Adriaens D, Adriaens P, Anselin A, Devos K, et al. (2011) Bridging the gap between the Natura 2000 regional conservation status and local conservation objectives. Journal for Nature Conservation 19: 224-235.

318. Lozano FD, Herbada DG, Rivero LM, Saiz JCM, Ollero HS (1996) Threatened plants in peninsular and balearic Spain: A report based on the EU habitats directive. Biological Conservation 76: 123-133.

319. Lukács BA, Sramkó G, Molnár V A (2013) Plant diversity and conservation value of continental temporary pools. Biological Conservation 158: 393-400.

320. Lumbreras A, Pardo C, Molina JA (2013) Bioindicator role of aquatic Ranunculus in Mediterranean freshwater habitats. Aquatic Conservation-Marine and Freshwater Ecosystems 23: 582-593.

321. Luoto M, Heikkinen RK, Carter TR (2004) Loss of palsa mires in Europe and biological consequences. Environmental Conservation 31: 30-37.

322. Luque EG, Mercado FG (2002) Rupicolous communities of the southeast of the Iberian Peninsula. Acta Botanica Gallica 149: 467-480.

323. Macedo-Sousa JA, Soares A, Tarazona JV (2009) A conceptual model for assessing risks in a Mediterranean Natura 2000 Network site. Science of the Total Environment 407: 1224-1231.

324. Maclean IMD, Austin GE, Rehfisch MM, Blew J, Crowe O, et al. (2008) Climate change causes rapid changes in the distribution and site abundance of birds in winter. Global Change Biology 14: 2489-2500.

325. Maes D, Collins S, Munguira ML, Sasic M, Settele J, et al. (2013) Not the Right Time to Amend the Annexes of the European Habitats Directive. Conservation Letters 6: 468-469.

326. Maes J, Paracchini ML, Zulian G, Dunbar MB, Alkemade R (2012) Synergies and trade-offs between ecosystem service supply, biodiversity, and habitat conservation status in Europe. Biological Conservation 155: 1-12.

327. Maffey G, Reed M, Irvine RJ, van der Wal R (2013) Habitat monitoring in the wider countryside: A case study on the pursuit of innovation in red deer management. Journal of Environmental Management 128: 779-786.

328. Maiorano L, Falcucci A, Garton EO, Boitani L (2007) Contribution of the Natura 2000 network to biodiversity conservation in Italy. Conservation Biology 21: 1433-1444.

329. Mairota P, Cafarelli B, Boccaccio L, Leronni V, Labadessa R, et al. (2013) Using landscape structure to develop quantitative baselines for protected area monitoring. Ecological Indicators 33: 82-95.

330. Malavasi M, Santoro R, Cutini M, Acosta ATR, Carranza ML (2013) What has happened to coastal dunes in the last half century? A multitemporal coastal landscape analysis in Central Italy. Landscape and Urban Planning 119: 54-63.

331. Mallard F, Francois D (2013) Effectiveness of the legal framework for natural areas protection relative to French road projects. Land Use Policy 30: 582-591.

332. Mallinis G, Emmanoloudis D, Giannakopoulos V, Maris F, Koutsias N (2011) Mapping and interpreting historical land cover/land use changes in a Natura 2000 site using earth observational data The case of Nestos delta, Greece. Applied Geography 31: 312-320.

333. Manzu C, Gherghel I, Zamfirescu S, Zamfirescu O, Rosca I, et al. (2013) Current And Future Potential Distribution Of Glacial Relict Ligularia sibirica (Asteraceae) In Romania And Temporal Contribution Of Natura 2000 To Protect The Species In Light Of Global Change. Carpathian Journal of Earth and Environmental Sciences 8: 77-87.

334. Marabuto E, Pires P, Corley MFV (2013) The Lepidoptera of Parque Natural do Tejo Internacional, Portugal (Insecta: Lepidoptera). Shilap-Revista De Lepidopterologia 41: 5-42.

335. Marage D, Gegout JC (2009) Importance of soil nutrients in the distribution of forest communities on a large geographical scale. Global Ecology and Biogeography 18: 88-97.

336. Marcer A, Garcia V, Escobar A, Pons X (2010) Handling historical information on protected-area systems and coverage. An information system for the Natura 2000 European context. Environmental Modelling & Software 25: 956-964.

337. Marignani M, Blasi C (2012) Looking for important plant areas: selection based on criteria, complementarity, or both? Biodiversity and Conservation 21: 1853-1864.

338. Marot N, Kolaric S, Golobic M (2013) Slovenia as the natural park of Europe? A territorial impact assessment in the case of Natura 2000. Acta Geographica Slovenica-Geografski Zbornik 53: 92-107.

339. Martincova J, Ondrasek L (2010) Grassland Monitoring of Meadows in the Region around Banska Bystrica. Czech Journal of Genetics and Plant Breeding 46: S40-S44.

340. Martinez I, Carreno F, Escudero A, Rubio A (2006) Are threatened lichen species well-protected in Spain? Effectiveness of a protected areas network. Biological Conservation 133: 500-511.

341. Martinez JE, Pagan I, Palazon JA, Calvo JF (2007) Habitat use of booted eagles (Hieraaetus pennatus) in a special protection area: Implications for conservation. Biodiversity and Conservation 16: 3481-3488.

342. Martinez-Hernandez F, Perez-Garcia FJ, Garrido-Becerra JA, Mendoza-Fernandez AJ, Medina-Cazorla JM, et al. (2011) The distribution of Iberian gypsophilous flora as a criterion for conservation policy. Biodiversity and Conservation 20: 1353-1364.

343. Marucco F, Boitani L (2012) Wolf population monitoring and livestock depredation preventive measures in Europe. Hystrix-Italian Journal of Mammalogy 23: 1-4.

344. Matern A, Desender K, Drees C, Gaublomme E, Paill WG, et al. (2009) Genetic diversity and population structure of the endangered insect species Carabus variolosus in its western distribution range: Implications for conservation. Conservation Genetics 10: 391-405.

345. Matern A, Drees C, Meyer H, Assmann T (2008) Population ecology of the rare carabid beetle Carabus variolosus (Coleoptera : Carabidae) in North-west Germany. Journal of Insect Conservation 12: 591-601.

346. Mazaris A, Tzanopoulos J, Kallimanis A, Matsinos Y, Sgardelis S, et al. (2008) The contribution of common and rare species to plant species richness patterns: the effect of habitat type and size of sampling unit. Biodiversity and Conservation 17: 3567-3577.

347. Mazaris AD, Kallimanis AS, Tzanopoulos J, Sgardelis SP, Pantis JD (2010) Can we predict the number of plant species from the richness of a few common genera, families or orders? Journal of Applied Ecology 47: 662-670.

348. Mazaris AD, Tsianou MA, Sigkounas A, Dimopoulos P, Pantis JD, et al. (2013) Accounting for the capacity of common and rare species to contribute to diversity spatial patterns: Is it a sampling issue or a biological effect? Ecological Indicators 32: 9-13.

349. Mazzei A, Bonacci T, Contarini E, Zetto T, Brandmayr P (2011) Rediscovering the 'umbrella species' candidate Cucujus cinnaberinus (Scopoli, 1763) in Southern Italy (Coleoptera Cucujidae), and notes on bionomy. Italian Journal of Zoology 78: 264-270.

350. McGillivray D (2012) Compensating Biodiversity Loss: The EU Commission's Approach to Compensation under Article 6 of the Habitats Directive. Journal of Environmental Law 24: 417-450.

351. McLaughlin E, Portig A, Johnson MP (2007) Can traditional harvesting methods for cockles be accommodated in a Special Area of Conservation? Ices Journal of Marine Science 64: 309-317.

352. Medina-Cazorla JM, de Carrasco CG, Merlo ME, Martinez-Hernandez F, Garrido-Becerra JA, et al. (2010) The dolomite shrublands of the Convolvuletalia boissieri order and their preservation by means of the Habitats Directive. Acta Botanica Gallica 157: 611-625.

353. Meireles C, Neiva R, Passos I, Vila-Vicosa C, Paiva-Ferreira R, et al. (2009) The management and preservation of communitarian interest habitats in the Natural Park of Serra da Estrela (Portugal). Acta Botanica Gallica 156: 79-88.

354. Meliadis I, Platis P, Ainalis A, Meliadis M (2010) Monitoring and analysis of natural vegetation in a Special Protected Area of Mountain Antichasia-Meteora, central Greece. Environmental Monitoring and Assessment 163: 455-465.

355. Mendes C, Dias E (2009) Characterisation of Sanguinhal Mire, Terceira Island (Azores): a protected quaking bog habitat. Acta Botanica Brasilica 23: 812-819.

356. Mendoza-Fernandez A, Perez-Garcia FJ, Medina-Cazorla JM, Martinez-Hernandez F, Garrido-Becerra JA, et al. (2010) Gap Analysis and selection of reserves for the threatened flora of eastern Andalusia, a hot spot in the eastern Mediterranean region. Acta Botanica Gallica 157: 749-767.

357. Merken R, Servaes F, Sfougaris A, Koedam N (2012) Birds in a complex agricultural landscape in Central Greece: the role of landscape elements and the landscape matrix. Journal of Biological Research-Thessaloniki 17: 137-147.

358. Metcalfe K, Roberts T, Smith RJ, Harrop SR (2013) Marine conservation science and governance in North-West Europe: Conservation planning and international law and policy. Marine Policy 39: 289-295.

359. Micael J, Alves MJ, Costa AC (2013) The population dynamics of Ophidiaster ophidianus (Echinodermata: Asteroidea) in the Azores, at the north-western periphery of its distribution. Journal of the Marine Biological Association of the United Kingdom 93: 1087-1095.

360. Michaelides G, Kati V (2009) Diversity patterns and conservation management of the lizard community in a Mediterranean reserve (Cyprus). Journal of Biological Research-Thessaloniki 12: 211-220.

361. Mikkelsen L, Mouritsen KN, Dahl K, Teilmann J, Tougaard J (2013) Re-established stony reef attracts harbour porpoises Phocoena phocoena. Marine Ecology Progress Series 481: 239-248.

362. Mikkonen N, Moilanen A (2013) Identification of top priority areas and management landscapes from a national Natura 2000 network. Environmental Science & Policy 27: 11-20.

363. Mikulcak F, Newig J, Milcu AI, Hartel T, Fischer J (2013) Integrating rural development and biodiversity conservation in Central Romania. Environmental Conservation 40: 129-137.

364. Milchev B, Georgiev V (2012) Roach's mouse-tailed dormouse Myomimus roachi distribution and conservation in Bulgaria. Hystrix-Italian Journal of Mammalogy 23: 66-70.

365. Milios E, Petrou P, Andreou E, Pipinis E (2011) Is facilitation the dominant process in the regeneration of the Juniperus excelsa M. Bieb. stands in Cyprus? Journal of Biological Research-Thessaloniki 16: 296-303.

366. Miller C (1997) Attributing 'priority' to habitats. Environmental Values 6: 341-353.

367. Millington JDA, Perry GLW, Romero-Calcerrada R (2007) Regression techniques for examining land use/cover change: A case study of a mediterranean landscape. Ecosystems 10: 562-578.

368. Mobaied S, Riera B, Lalanne A, Baguette M, Machon N (2011) The use of diachronic spatial approaches and predictive modelling to study the vegetation dynamics of a managed heathland. Biodiversity and Conservation 20: 73-88.

369. Moeslund JE, Arge L, Bocher PK, Dalgaard T, Ejrnaes R, et al. (2013) Topographically controlled soil moisture drives plant diversity patterns within grasslands. Biodiversity and Conservation 22: 2151-2166.

370. Moeslund JE, Arge L, Bocher PK, Nygaard B, Svenning JC (2011) Geographically Comprehensive Assessment of Salt-Meadow Vegetation-Elevation Relations Using LiDAR. Wetlands 31: 471-482.

371. Mony C, Mony JF, Thiebaut G, Muller S (2006) Floristic and ecological diversity of Ranunculus aquatic habitats in the sub-Atlantic range: implications for conservation. Biodiversity and Conservation 15: 3383-3400.

372. Moran-Ordonez A, Suarez-Seoane S, Elith J, Calvo L, de Luis E (2012) Satellite surface reflectance improves habitat distribution mapping: a case study on heath and shrub formations in the Cantabrian Mountains (NW Spain). Diversity and Distributions 18: 588-602.

373. Moreno V, Morales MB, Traba J (2010) Avoiding over-implementation of agri-environmental schemes for steppe bird conservation: A species-focused proposal based on expert criteria. Journal of Environmental Management 91: 1802-1809.

374. Morris RKA (2011) The application of the Habitats Directive in the UK: Compliance or gold plating? Land Use Policy 28: 361-369.

375. Morsing J, Frandsen SI, Vejre H, Raulund-Rasmussen K (2013) Do the Principles of Ecological Restoration Cover EU LIFE Nature Cofunded Projects in Denmark? Ecology and Society 18: 13.

376. Mossman HL, Davy AJ, Grant A (2012) Does managed coastal realignment create saltmarshes with 'equivalent biological characteristics' to natural reference sites? Journal of Applied Ecology 49: 1446-1456.

377. Mouro C, Castro P (2010) Local Communities Responding to Ecological Challenges-A Psycho-social Approach to the Natura 2000 Network. Journal of Community & Applied Social Psychology 20: 139-155.

378. Mucher CA, Hennekens SM, Bunce RGH, Schaminee JHJ, Schaepman ME (2009) Modelling the spatial distribution of Natura 2000 habitats across Europe. Landscape and Urban Planning 92: 148-159.

379. Mucher CA, Kooistra L, Vermeulen M, Vanden Borre J, Haest B, et al. (2013) Quantifying structure of Natura 2000 heathland habitats using spectral mixture analysis and segmentation techniques on hyperspectral imagery. Ecological Indicators 33: 71-81.

380. Muller J, Brunet J, Brin A, Bouget C, Brustel H, et al. (2013) Implications from large-scale spatial diversity patterns of saproxylic beetles for the conservation of European Beech forests. Insect Conservation and Diversity 6: 162-169.

381. Muller J, Gossner MM (2010) Three-dimensional partitioning of diversity informs state-wide strategies for the conservation of saproxylic beetles. Biological Conservation 143: 625-633.

382. Muller J, Pollath J, Moshammer R, Schroder B (2009) Predicting the occurrence of Middle Spotted Woodpecker Dendrocopos medius on a regional scale, using forest inventory data. Forest Ecology and Management 257: 502-509.

383. Muller S (2002) Appropriate agricultural management practices required to ensure conservation and biodiversity of environmentally sensitive grassland sites designated under Natura 2000. Agriculture Ecosystems & Environment 89: 261-266.

384. Murray TE, Fitzpatrick U, Byrne A, Fealy R, Brown MJF, et al. (2012) Local-scale factors structure wild bee communities in protected areas. Journal of Applied Ecology 49: 998-1008.

385. Nascimbene J, Benesperi R, Brunialti G, Catalano I, Vedove MD, et al. (2013) Patterns and drivers of beta-diversity and similarity of Lobaria pulmonaria communities in Italian forests. Journal of Ecology 101: 493-505.

386. Nascimbene J, Marini L, Nimis PL (2010) Epiphytic lichen diversity in old-growth and managed Picea abies stands in Alpine spruce forests. Forest Ecology and Management 260: 603-609.

387. Nascimbene J, Nimis PL, Ravera S (2013) Evaluating the conservation status of epiphytic lichens of Italy: A red list. Plant Biosystems 147: 898-904.

388. Ni Bhriain B, Skeffington MS, Gormally M (2002) Conservation implications of land use practices on the plant and carabid beetle communities of two turloughs in Co. Galway, Ireland. Biological Conservation 105: 81-92.

389. Niedzialkowski K, Paavola J, Jedrzejewska B (2013) Governance of biodiversity in Poland before and after the accession to the EU: the tale of two roads. Environmental Conservation 40: 108-118.

390. Normand S, Svenning JC, Skov F (2007) National and European perspectives on climate change sensitivity of the habitats directive characteristic plant species. Journal for Nature Conservation 15: 41-53.

391. Nunez V, Hernando A, Velazquez J, Tejera R (2012) Livestock management in Natura 2000: A case study in a Quercus pyrenaica neglected coppice forest. Journal for Nature Conservation 20: 1-9.

392. Obidzinski A, Kloss M, Cedro A (2009) Is spontaneous regeneration of raised mire vegetation possible? A case study of the 'Czarne Bagno' mire in the Bystrzyckie Hills, southern Poland. Holocene 19: 229-239.

393. O'Brien SH, Webb A, Brewer MJ, Reid JB (2012) Use of kernel density estimation and maximum curvature to set Marine Protected Area boundaries: Identifying a Special Protection Area for wintering red-throated divers in the UK. Biological Conservation 156: 15-21.

394. Oikonomakis N, Ganatsas P (2012) Land cover changes and forest succession trends in a site of Natura 2000 network (Elatia forest), in northern Greece. Forest Ecology and Management 285: 153-163.

395. Oikonomou V, Dimitrakopoulos PG, Troumbis AY (2011) Incorporating Ecosystem Function Concept in Environmental Planning and Decision Making by Means of Multi-Criteria Evaluation: The Case-Study of Kalloni, Lesbos, Greece. Environmental Management 47: 77-92.

396. Olsson PA, Martensson LM, Bruun HH (2009) Acidification of sandy grasslands - consequences for plant diversity. Applied Vegetation Science 12: 350-361.

397. Opdam PFM, Broekmeyer MEA, Kistenkas FH (2009) Identifying uncertainties in judging the significance of human impacts on Natura 2000 sites. Environmental Science & Policy 12: 912-921.

398. Opermanis O, MacSharry B, Aunins A, Sipkova Z (2012) Connectedness and connectivity of the Natura 2000 network of protected areas across country borders in the European Union. Biological Conservation 153: 227-238.

399. Opermanis O, MacSharry B, Evans D, Sipkova Z (2013) Is the connectivity of the Natura 2000 network better across internal or external administrative borders? Biological Conservation 166: 170-174.

400. Ostendorp W, Gretler T, Mainberger M, Peintinger M, Schmieder K (2009) Effects of mooring management on submerged vegetation, sediments and macro-invertebrates in Lake Constance, Germany. Wetlands Ecology and Management 17: 525-541.

401. Ostermann OP (1998) The need for management of nature conservation sites designated under Natura 2000. Journal of Applied Ecology 35: 968-973.

402. Oszlanyi J, Grodzinska K, Badea O, Shparyk Y (2004) Nature conservation in Central and Eastern Europe with a special emphasis on the Carpathian Mountains. Environmental Pollution 130: 127-134.

403. Otegui J, Villarroya A, Arino AH (2012) Protected areas in the Spanish Pyrenees: A meaningful way to preserve biodiversity? Environmental Engineering and Management Journal 11: 1133-1140.

404. Paal J (2009) The forests of the North-Estonian Klint; the north-easternmost representatives of the EU Habitat Directive Tilio-Acerion forests of slopes, screes and ravines. Annales Botanici Fennici 46: 525-540.

405. Palo A, Ivask M, Liira J (2013) Biodiversity composition reflects the history of ancient semi-natural woodland and forest habitats-Compilation of an indicator complex for restoration practice. Ecological Indicators 34: 336-344.

406. Panitsa M, Koutsias N, Tsiripidis I, Zotos A, Dimopoulos P (2011) Species-based versus habitat-based evaluation for conservation status assessment of habitat types in the East Aegean islands (Greece). Journal for Nature Conservation 19: 269-275.

407. Papageorgiou K, Vogiatzakis IN (2006) Nature protection in Greece: an appraisal of the factors shaping integrative conservation and policy effectiveness. Environmental Science & Policy 9: 476-486.

408. Papaioannou HI, Kati VI (2007) Current status of the Balkan chamois (Rupicapra rupicapra balcanica) in Greece: Implications for conservation. Belgian Journal of Zoology 137: 33-39.

409. Papanikolaou AD, Fyllas NM, Mazaris AD, Dimitrakopoulos PG, Kallimanis AS, et al. (2011) Grazing effects on plant functional group diversity in Mediterranean shrublands. Biodiversity and Conservation 20: 2831-2843.

410. Papastergiadou E, Kagalou I, Stefanidis K, Retalis A, Leonardos I (2010) Effects of Anthropogenic Influences on the Trophic State, Land Uses and Aquatic Vegetation in a Shallow Mediterranean Lake: Implications for Restoration. Water Resources Management 24: 415-435.

411. Parolo G, Abeli T, Gusmeroli F, Rossi G (2011) Large-scale heterogeneous cattle grazing affects plant diversity and forage value of Alpine species-rich Nardus pastures. Grass and Forage Science 66: 541-550.

412. Parolo G, Ferrarini A, Rossi G (2009) Optimization of tourism impacts within protected areas by means of genetic algorithms. Ecological Modelling 220: 1138-1147.

413. Pascual LL, Maiorano L, Alessandra F, Emilio B, Luigi B (2011) Hotspots of species richness, threat and endemism for terrestrial vertebrates in SW Europe. Acta Oecologica-International Journal of Ecology 37: 399-412.

414. Pedersen SA, Fock H, Krause J, Pusch C, Sell AL, et al. (2009) Natura 2000 sites and fisheries in German offshore waters. Ices Journal of Marine Science 66: 155-169.

415. Pedersen SA, Fock HO, Sell AF (2009) Mapping fisheries in the German exclusive economic zone with special reference to offshore Natura 2000 sites. Marine Policy 33: 571-590.

416. Pellissier V, Touroult J, Julliard R, Siblet JP, Jiguet F (2013) Assessing the Natura 2000 network with a common breeding birds survey. Animal Conservation 16: 566-574.

417. Perez-Hugalde C, Romero-Calcerrada R, Delgado-Perez P, Novillo CJ (2011) Understanding land cover change in a Special Protection Area in Central Spain through the enhanced land cover transition matrix and a related new approach. Journal of Environmental Management 92: 1128-1137.

418. Perrino EV, Tomaselli V, Costa R, Pavone P (2013) Conservation status of habitats (Directive 92/43 EEC) of coastal and low hill belts in a Mediterranean biodiversity hot spot (Gargano - Italy). Plant Biosystems 147: 1006-1028.

419. Petrosillo I, Semeraro T, Zurlini G (2010) Detecting the 'conservation effect' on the maintenance of natural capital flow in different natural parks. Ecological Economics 69: 1115-1123.

420. Petrosillo I, Zaccarelli N, Semeraro T, Zurlini G (2009) The effectiveness of different conservation policies on the security of natural capital. Landscape and Urban Planning 89: 49-56.

421. Pietrzyk-Kaszynska A, Cent J, Grodzinska-Jurczak M, Szymanska M (2012) Factors influencing perception of protected areas-The case of Natura 2000 in Polish Carpathian communities. Journal for Nature Conservation 20: 284-292.

422. Pinto B, Partidario M (2012) The History of the Establishment and Management Philosophies of the Portuguese Protected Areas: Combining Written Records and Oral History. Environmental Management 49: 788-801.

423. Pinto-Cruz C, Barbosa AM, Molina JA, Espirito-Santo MD (2011) Biotic and abiotic parameters that distinguish types of temporary ponds in a Portuguese Mediterranean ecosystem. Ecological Indicators 11: 1658-1663.

424. Pinto-Cruz C, Molina JA, Barbour M, Silva V, Espirito-Santo MD (2009) Plant communities as a tool in temporary ponds conservation in SW Portugal. Hydrobiologia 634: 11-24.

425. Piorr A, Ungaro F, Ciancaglini A, Happe K, Sahrbacher A, et al. (2009) Integrated assessment of future CAP policies: land use changes, spatial patterns and targeting. Environmental Science & Policy 12: 1122-1136.

426. Piquer-Rodriguez M, Kuemmerle T, Alcaraz-Segura D, Zurita-Milla R, Cabello J (2012) Future land use effects on the connectivity of protected area networks in southeastern Spain. Journal for Nature Conservation 20: 326-336.

427. Pisanu S, Filigheddu R, Farris E (2009) The conservation status of an endemic species of northern Sardinia: Centaurea horrida Badaro (Asteraceae). Plant Biosystems 143: 275-282.

428. Platteeuw M, Foppen RPB, van Eerden MR (2010) The need for future wetland bird studies: scales of habitat use as input for ecological restoration and spatial water management. Ardea 98: 403-416.

429. Poirazidis K, Schindler S, Kakalis E, Ruiz C, Bakaloudis DE, et al. (2011) Population Estimates For The Diverse Raptor Assemblage Of Dadia National Park, Greece. Ardeola 58: 3-17.

430. Pokluda P, Hauck D, Cizek L (2012) Importance of marginal habitats for grassland diversity: fallows and overgrown tall-grass steppe as key habitats of endangered ground-beetle Carabus hungaricus. Insect Conservation and Diversity 5: 27-36.

431. Porst G, Irvine K (2009) Implications of the spatial variability of macroinvertebrate communities for monitoring of ephemeral lakes. An example from turloughs. Hydrobiologia 636: 421-438.

432. Porst G, Irvine K (2009) Distinctiveness of macroinvertebrate communities in turloughs (temporary ponds) and their response to environmental variables. Aquatic Conservation-Marine and Freshwater Ecosystems 19: 456-465.

433. Prajs B, Antkowiak W (2010) Grassland Ecosystems in the Varied Hydrological and Ecological Conditions of the Kulawa River Valley. Polish Journal of Environmental Studies 19: 131-139.

434. Prazan J, Ratinger T, Krumalova V (2005) The evolution of nature conservation policy in the Czech Republic - challenges of europeanisation in the White Carpathians Protected Landscape Area. Land Use Policy 22: 235-243.

435. Pröbstl U (2003) NATURA 2000 - The influence of the European directives on the development of nature-based sport and outdoor recreation in mountain areas. Journal for Nature Conservation 11: 340-345.

436. Puente A, Juanes JA (2008) Testing taxonomic resolution, data transformation and selection of species for monitoring macroalgae communities. Estuarine Coastal and Shelf Science 78: 327-340.

437. Pullin AS, Baldi A, Can OE, Dieterich M, Kati V, et al. (2009) Conservation Focus on Europe: Major Conservation Policy Issues That Need to Be Informed by Conservation Science. Conservation Biology 23: 818-824.

438. Rainho A, Palmeirim JM (2013) Prioritizing conservation areas around multispecies bat colonies using spatial modeling. Animal Conservation 16: 438-448.

439. Ramirez JA, Diaz M (2008) The role of temporal shrub encroachment for the maintenance of Spanish holm oak Quercus ilex dehesas. Forest Ecology and Management 255: 1976-1983.

440. Ranius T (2002) Osmoderma eremita as an indicator of species richness of beetles in tree hollows. Biodiversity and Conservation 11: 931-941.

441. Regan EC, Skeffington MS, Gormally MJ (2007) Wetland plant communities of turloughs in southeast Galway/north Clare, Ireland in relation to environmental factors. Aquatic Botany 87: 22-30.

442. Reid N, Dingerkus SK, Stone RE, Pietravalle S, Kelly R, et al. (2013) Population enumeration and assessing conservation status in a widespread amphibian: a case study of Rana temporaria in Ireland. Animal Conservation 16: 519-527.

443. Reid N, Keys A, Preston JS, Moorkens E, Roberts D, et al. (2013) Conservation status and reproduction of the critically endangered freshwater pearl mussel (Margaritifera margaritifera) in Northern Ireland. Aquatic Conservation-Marine and Freshwater Ecosystems 23: 571-581.

444. Reid N, Lundy MG, Hayden B, Lynn D, Marnell F, et al. (2013) Detecting detectability: identifying and correcting bias in binary wildlife surveys demonstrates their potential impact on conservation assessments. European Journal of Wildlife Research 59: 869-879.

445. Rekola M, Pouta E, Kuuluvainen J, Tahvonen O, Li CZ (2000) Incommensurable preferences in contingent valuation: the case of Natura 2000 Network in Finland. Environmental Conservation 27: 260-268.

446. Ribeiro S, Ladero M, Espirito-Santo MD (2013) Patterns of floristic composition of Mediterranean meadows and mesophytic grasslands in eastern Continental Portugal. Plant Biosystems 147: 874-892.

447. Rodder D, Schulte U (2010) Potential loss of genetic variability despite well established network of reserves: the case of the Iberian endemic lizard Lacerta schreiberi. Biodiversity and Conservation 19: 2651-2666.

448. Roder D, Kiehl K (2006) Population structure and population dynamic of Pulsatilla patens (L.) Mill. in relation to vegetation characteristics. Flora 201: 499-507.

449. Rodriguez-Oubina J, Izco J, Ramil P (2001) Phytosociological characterization of Sphagnum pylaesii Brid. communities in Northwest Spain. Acta Botanica Gallica 148: 201-213.

450. Roeder N, Lederbogen D, Trautner J, Bergamini A, Stofer S, et al. (2010) The impact of changing agricultural policies on jointly used rough pastures in the Bavarian Pre-Alps: An economic and ecological scenario approach. Ecological Economics 69: 2435-2447.

451. Romero MI, Ramil P, Rubinos M (2004) Conservation status of Eryngium viviparum Gay. Acta Botanica Gallica 151: 55-64.

452. Rosa HD, Da Silva JM (2005) From environmental ethics to nature conservation policy: Natura 2000 and the burden of proof. Journal of Agricultural & Environmental Ethics 18: 107-130.

453. Rosati L, Marignani M, Blasi C (2008) A gap analysis comparing Natura 2000 vs National Protected Area network with potential natural vegetation. Community Ecology 9: 147-154.

454. Rosen T, Bath A (2009) Transboundary management of large carnivores in Europe: from incident to opportunity. Conservation Letters 2: 109-114.

455. Rowell TA (2009) Management planning guidance for protected sites in the UK; a comparison of formats and definitions in nine guides. Journal for Nature Conservation 17: 99-111.

456. Rozbrojova Z, Hajek M, Hajek O (2010) Vegetation diversity of mesic meadows and pastures in the West Carpathians. Preslia 82: 307-332.

457. Rubio-Salcedo M, Martinez I, Carreno F, Escudero A (2013) Poor effectiveness of the Natura 2000 network protecting Mediterranean lichen species. Journal for Nature Conservation 21: 1-9.

458. Rudner M (2011) Ephemeral Wetland Vegetation in Mediterranean Heathland and Maquis Communities. Wetlands 31: 551-562.

459. Russo P, Carullo L, Riguccio L, Tomaselli G (2011) Identification of landscapes for drafting Natura 2000 network Management Plans: A case study in Sicily. Landscape and Urban Planning 101: 228-243.

460. Ryder C, Moran J, Mc Donnell R, Gormally M (2005) Conservation implications of grazing practices on the plant and dipteran communities of a turlough in Co. Mayo, Ireland. Biodiversity and Conservation 14: 187-204.

461. Saiz JCM, Lozano FD, Ollero HS (2003) Recent progress in conservation of threatened Spanish vascular flora: a critical review. Biological Conservation 113: 419-431.

462. Salvatori V, Mertens AD (2012) Damage prevention methods in Europe: experiences from LIFE nature projects. Hystrix-Italian Journal of Mammalogy 23: 73-79.

463. Sanchez-Fernandez D, Abellan P, Picazo F, Millan A, Ribera I, et al. (2013) Do protected areas represent species' optimal climatic conditions? A test using Iberian water beetles. Diversity and Distributions 19: 1407-1417.

464. Sanchez-Fernandez D, Abellan P, Velasco J, Millan A (2004) Selecting areas to protect the biodiversity of aquatic ecosystems in a semiarid Mediterranean region using water beetles. Aquatic Conservation-Marine and Freshwater Ecosystems 14: 465-479.

465. Sanchez-Fernandez D, Bilton DT, Abellan P, Ribera I, Vezasco J, et al. (2008) Are the endemic water beetles of the Iberian Peninsula and the Balearic Islands effectively protected? Biological Conservation 141: 1612-1627.

466. Sanchez-Hernandez C, Boyd DS, Foody GM (2007) Mapping specific habitats from remotely sensed imagery: Support vector machine and support vector data description based classification of coastal saltmarsh habitats. Ecological Informatics 2: 83-88.

467. Sandor AD, Domsa C (2012) Special Protected Areas for Conservation of Romania' Forest Birds: Status Assessment and Possible Expansion using Predictive Tools. Acta Zoologica Bulgarica 64: 367-374.

468. Santangeli A, Wistbacka R, Hanski IK, Laaksonen T (2013) Ineffective enforced legislation for nature conservation: A case study with Siberian flying squirrel and forestry in a boreal landscape. Biological Conservation 157: 237-244.

469. Santi E, Mari E, Piazzini S, Renzi M, Bacaro G, et al. (2010) Dependence of animal diversity on plant diversity and environmental factors in farmland ponds. Community Ecology 11: 232-241.

470. Santoro R, Carboni M, Carranza ML, Acosta ATR (2012) Focal species diversity patterns can provide diagnostic information on plant invasions. Journal for Nature Conservation 20: 85-91.

471. Santos KC, Pino J, Roda F, Guirado M, Ribas J (2008) Beyond the reserves: The role of non-protected rural areas for avifauna conservation in the area of Barcelona (NE of Spain). Landscape and Urban Planning 84: 140-151.

472. Sarvasova Z, Salka J, Dobsinska Z (2013) Mechanism of cross-sectoral coordination between nature protection and forestry in the Natura 2000 formulation process in Slovakia. Journal of Environmental Management 127: S65-S72.

473. Sastre P, Ponce C, Palacin C, Martin CA, Alonso JC (2009) Disturbances to great bustards (Otis tarda) in central Spain: human activities, bird responses and management implications. European Journal of Wildlife Research 55: 425-432.

474. Saura S, Pascual-Hortal L (2007) A new habitat availability index to integrate connectivity in landscape conservation planning: Comparison with existing indices and application to a case study. Landscape and Urban Planning 83: 91-103.

475. Schaefer H, Carine MA, Rumsey FJ (2011) From european priority species to invasive weed: Marsilea azorica (Marsileaceae) is a misidentified alien. Systematic Botany 36: 845-853.

476. Schmeller D, Gruber B, Bauch B, Lanno K, Budrys E, et al. (2008) Determination of national conservation responsibilities for species conservation in regions with multiple political jurisdictions. Biodiversity and Conservation 17: 3607-3622.

477. Schmieder K (2004) European lake shores in danger - concepts for a sustainable development. Limnologica 34: 3-14.

478. Schnittler M, Gunther KF (1999) Central European vascular plants requiring priority conservation measures - an analysis from national Red Lists and distribution maps. Biodiversity and Conservation 8: 891-925.

479. Sebastia MT, Rodilla M, Sanchis JA, Altur V, Gadea I, et al. (2012) Influence of nutrient inputs from a wetland dominated by agriculture on the phytoplankton community in a shallow harbour at the Spanish Mediterranean coast. Agriculture Ecosystems & Environment 152: 10-20.

480. Selva N, Kreft S, Kati V, Schluck M, Jonsson BG, et al. (2011) Roadless and low-traffic reas as conservation targets in Europe. Environmental Management 48: 865-877.

481. Selvi F, Valleri M (2012) Cork oak woodlands in the north Tyrrhenian area (Italy): distribution and plant species diversity of a relict forest ecosystem. Biodiversity and Conservation 21: 3061-3078.

482. Seoane J, Justribo JH, Garcia F, Retamar J, Rabadan C, et al. (2006) Habitat-suitability modelling to assess the effects of land-use changes on Dupont's lark Chersophilus duponti: A case study in the Layna Important Bird Area. Biological Conservation 128: 241-252.

483. Simonson WD, Allen HD, Coomes DA (2012) Use of an Airborne Lidar System to Model Plant Species Composition and Diversity of Mediterranean Oak Forests. Conservation Biology 26: 840-850.

484. Simonson WD, Allen HD, Coomes DA (2013) Remotely sensed indicators of forest conservation status: Case study from a Natura 2000 site in southern Portugal. Ecological Indicators 24: 636-647.

485. Skeffington MS, Gormally M (2007) Turloughs: A mosaic of biodiversity and management systems unique to Ireland. Acta Carsologica 36: 217-222.

486. Skeffington MS, Moran J, O Connor A, Regan E, Coxon CE, et al. (2006) Ttirloughs - Ireland's unique wetland habitat. Biological Conservation 133: 265-290.

487. Skorka P, Settele J, Woyciechowski M (2007) Effects of management cessation on grassland butterflies in southern Poland. Agriculture Ecosystems & Environment 121: 319-324.

488. Slepcevic R (2009) The judicial enforcement of EU law through national courts: possibilities and limits. Journal of European Public Policy 16: 378-394.

489. Slepetiene A, Liaudanskiene I, Slepetys J, Stukonis V, Jokubauskaite I, et al. (2013) Comparison of soil organic matter content and composition in protected areas of NATURA 2000 and agrarian lands. Journal of Food Agriculture & Environment 11: 1105-1110.

490. Soane ID, Scolozzi R, Gretter A, Hubacek K (2012) Exploring Panarchy in Alpine Grasslands: an Application of Adaptive Cycle Concepts to the Conservation of a Cultural Landscape. Ecology and Society 17: 11.

491. Solano E, Mancini E, Ciucci P, Mason F, Audisio P, et al. (2013) The EU protected taxon Morimus funereus Mulsant, 1862 (Coleoptera: Cerambycidae) and its western Palaearctic allies: systematics and conservation outcomes. Conservation Genetics 14: 683-694.

492. Spanhove T, Vanden Borre J, Delalieux S, Haest B, Paelinckx D (2012) Can remote sensing estimate fine-scale quality indicators of natural habitats? Ecological Indicators 18: 403-412.

493. Spitzer L, Benes J, Dandova J, Jaskova V, Konvicka M (2009) The Large Blue butterfly, Phengaris Maculinea arion, as a conservation umbrella on a landscape scale: The case of the Czech Carpathians. Ecological Indicators 9: 1056-1063.

494. Stancioiu PT, Abrudan IV, Dutca I (2010) The Natura 2000 ecological network and forests in Romania: implications on management and administration. International Forestry Review 12: 106-113.

495. Stechova T, Hajek M, Hajkova P, Navratilova J (2008) Comparison of habitat requirements of the mosses Hamatocaulis vernicosus, Scorpidium cossonii and Warnstorfia exannulata in different parts of temperate Europe. Preslia 80: 399-410.

496. Stillman RA, Moore JJ, Woolmer AP, Murphy MD, Walkere P, et al. (2010) Assessing waterbird conservation objectives: An example for the Burry Inlet, UK. Biological Conservation 143: 2617-2630.

497. Stloukal E, Harvanekova M (2005) Distribution of Austropotamobius torrentium (Decapoda : Astacidae) in Slovakia. Bulletin Francais De La Peche Et De La Pisciculture: 547-552.

498. Stone EL, Jones G, Harris S (2013) Mitigating the Effect of Development on Bats in England with Derogation Licensing. Conservation Biology 27: 1324-1334.

499. Strange N, Jacobsen JB, Thorsen BJ, Tarp P (2007) Value for money: Protecting endangered species on Danish heathland. Environmental Management 40: 761-774.

500. Stringell TB, Bamber RN, Burton M, Lindenbaum C, Skates LR, et al. (2013) A tool for protected area management: multivariate control charts "cope' with rare variable communities. Ecology and Evolution 3: 1667-1676.

501. Stringer LC, Paavola J (2013) Participation in environmental conservation and protected area management in Romania: A review of three case studies. Environmental Conservation 40: 138-146.

502. Sullivan CA, Bourke D, Skeffington MS, Finn JA, Green S, et al. (2011) Modelling semi-natural habitat area on lowland farms in western Ireland. Biological Conservation 144: 1089-1099.

503. Sumares D, Fidelis T (2009) Local perceptions and postures towards the SPA "Ria de Aveiro". Journal of Integrative Environmental Sciences 6: 121-137.

504. Sumares D, Fidelis T (2011) Natura 2000 and the narrative nature of nature: a case for critical discourse analysis. Journal of Integrative Environmental Sciences 8: 53-68.

505. Sundblad G, Bergstrom U, Sandstrom A (2011) Ecological coherence of marine protected area networks: a spatial assessment using species distribution models. Journal of Applied Ecology 48: 112-120.

506. Sveegaard S, Teilmann J, Berggren P, Mouritsen KN, Gillespie D, et al. (2011) Acoustic surveys confirm the high-density areas of harbour porpoises found by satellite tracking. Ices Journal of Marine Science 68: 929-936.

507. Szymura TH, Szymura M (2013) Spatial variability more influential than soil pH and land relief on thermophilous vegetation in overgrown coppice oak forests. Acta Societatis Botanicorum Poloniae 82: 5-11.

508. Szymura TH, Szymura M, Pietrzak M (2014) Influence of land relief and soil properties on stand structure of overgrown oak forests of coppice origin with Sorbus torminalis. Dendrobiology 71: 49-58.

509. Tattoni C, Ciolli M, Ferretti F (2011) The fate of priority areas for conservation in protected areas: A fine-scale Markov chain approach. Environmental Management 47: 263-278.

510. Teofili C, Battisti C (2011) May the Conservation Measures Partnership open standards framework improve the effectiveness of the Natura 2000 European Network? A comparative analysis. Journal of Integrative Environmental Sciences 8: 7-21.

511. Therivel R (2009) Appropriate assessment of plans in England. Environmental Impact Assessment Review 29: 261-272.

512. Thomaes A, Kervyn T, Maes D (2008) Applying species distribution modelling for the conservation of the threatened saproxylic Stag Beetle (Lucanus cervus). Biological Conservation 141: 1400-1410.

513. Thompson PM, Hastie GD, Nedwell J, Barham R, Brookes KL, et al. (2013) Framework for assessing impacts of pile-driving noise from offshore wind farm construction on a harbour seal population. Environmental Impact Assessment Review 43: 73-85.

514. Thompson PM, Mackey B, Barton TR, Duck C, Butler JRA (2007) Assessing the potential impact of salmon fisheries management on the conservation status of harbour seals (Phoca vitulina) in north-east Scotland. Animal Conservation 10: 48-56.

515. Thompson PM, Van Parijs S, Kovacs KM (2001) Local declines in the abundance of harbour seals: implications for the designation and monitoring of protected areas. Journal of Applied Ecology 38: 117-125.

516. Thoonen G, Hufkens K, Vanden Borre J, Spanhove T, Scheunders P (2012) Accuracy assessment of contextual classification results for vegetation mapping. International Journal of Applied Earth Observation and Geoinformation 15: 7-15.

517. Tomankova I, Boland H, Reid N, Fox AD (2013) Assessing the extent to which temporal changes in waterbird community composition are driven by either local, regional or global factors. Aquatic Conservation-Marine and Freshwater Ecosystems 23: 343-355.

518. Tomaselli V, Dimopoulos P, Marangi C, Kallimanis AS, Adamo M, et al. (2013) Translating land cover/land use classifications to habitat taxonomies for landscape monitoring: a Mediterranean assessment. Landscape Ecology 28: 905-930.

519. Tomaselli V, Tenerelli P, Sciandrello S (2012) Mapping and quantifying habitat fragmentation in small coastal areas: a case study of three protected wetlands in Apulia (Italy). Environmental Monitoring and Assessment 184: 693-713.

520. Tonder M, Jurvelius J (2004) Attitudes towards fishery and conservation of the Saimaa ringed seal in Lake Pihlajavesi, Finland. Environmental Conservation 31: 122-129.

521. Torbidoni EIF (2011) Managing for Recreational Experience Opportunities: The Case of Hikers in Protected Areas in Catalonia, Spain. Environmental Management 47: 482-496.

522. Trigas P, Tsiftsis S, Tsiripidis I, Iatrou G (2012) Distribution patterns and conservation perspectives of the endemic flora of Peloponnese (Greece). Folia Geobotanica 47: 421-439.

523. Trivedi MR, Morecroft MD, Berry PM, Dawson TP (2008) Potential effects of climate change on plant communities in three montane nature reserves in Scotland, UK. Biological Conservation 141: 1665-1675.

524. Trouwborst A (2010) Managing the Carnivore Comeback: International and EU Species Protection Law and the Return of Lynx, Wolf and Bear to Western Europe. Journal of Environmental Law 22: 347-372.

525. Tryjanowski P, Hartel T, Baldi A, Szymanski P, Tobolka M, et al. (2011) Conservation of farmland birds faces different challenges in Western and Central-Eastern Europe. Acta Ornithologica 46: 1-12.

526. Tsiafouli MA, Apostolopoulou E, Mazaris AD, Kallimanis AS, Drakou EG, et al. (2013) Human Activities in Natura 2000 Sites: A Highly Diversified Conservation Network. Environmental Management 51: 1025-1033.

527. Tsianou MA, Mazaris AD, Kallimanis AS, Deligioridi PSK, Apostolopoulou E, et al. (2013) Identifying the criteria underlying the political decision for the prioritization of the Greek Natura 2000 conservation network. Biological Conservation 166: 103-110.

528. Tsiftsis S, Tsiripidis I, Karagiannakidou V (2009) Identifying areas of high importance for orchid conservation in east Macedonia (NE Greece). Biodiversity and Conservation 18: 1765-1780.

529. Van der Lee GEM, Van der Molen DT, Van den Boogaard HFP, Van der Klis H (2006) Uncertainty analysis of a spatial habitat suitability model and implications for ecological management of water bodies. Landscape Ecology 21: 1019-1032.

530. Vanden Borre J, Paelinckx D, Mucher CA, Kooistra L, Haest B, et al. (2011) Integrating remote sensing in Natura 2000 habitat monitoring: Prospects on the way forward. Journal for Nature Conservation 19: 116-125.

531. Vanderpoorten A, Sotiaux A, Engels P (2005) A GIS-based survey for the conservation of bryophytes at the landscape scale. Biological Conservation 121: 189-194.

532. Vardinoyannis K, Demetropoulos S, Mylonas M, Triantis KA, Makris C, et al. (2012) Terrestrial slugs (Gastropoda, Pulmonata) in the NATURA 2000 areas of Cyprus island. Zookeys: 63-77.

533. Varela RAD, Rego PR, Iglesias SC, Sobrino CM (2008) Automatic habitat classification methods based on satellite images: A practical assessment in the NW Iberia coastal mountains. Environmental Monitoring and Assessment 144: 229-250.

534. Veen P, Fanta J, Raev I, Biris IA, de Smidt J, et al. (2010) Virgin forests in Romania and Bulgaria: results of two national inventory projects and their implications for protection. Biodiversity and Conservation 19: 1805-1819.

535. Vela E, Auda P, Leger JF, Goncalves V, Baumel A (2008) Example of a new evaluation of threat status according to the IUCN criteria 3.1: the case of the Provencal endemic Arenaria provincialls Chater & Halliday (Caryophyllaceae). Acta Botanica Gallica 155: 547-562.

536. Velazquez J, Tejera R, Hernando A, Nunez MV (2010) Environmental diagnosis: Integrating biodiversity conservation in management of Natura 2000 forest spaces. Journal for Nature Conservation 18: 309-317.

537. Vellak A, Tuvi EL, Reier U, Kalamees R, Roosaluste E, et al. (2009) Past and Present Effectiveness of Protected Areas for Conservation of Naturally and Anthropogenically Rare Plant Species. Conservation Biology 23: 750-757.

538. Venturas M, Alvarez SG, Alcantara MF, Collada C, Gil L (2013) Species selection for reforestations: what happens with historic local extinctions and habitat protection zones? A case study in the Cantabrian Range. European Journal of Forest Research 132: 107-120.

539. Verovnik R, Govedic M, Salamun A (2011) Is the Natura 2000 network sufficient for conservation of butterfly diversity? A case study in Slovenia. Journal of Insect Conservation 15: 345-350.

540. Vikolainen V, Bressers H, Lulofs K (2013) The role of Natura 2000 and project design in implementing flood defence projects in the Scheldt estuary. Journal of Environmental Planning and Management 56: 1359-1379.

541. Vink SN, Neilson R, Robinson D, Daniell TJ (2014) Temporal and land use effects on soil bacterial community structure of the machair, an EU Habitats Directive Annex I low-input agricultural system. Applied Soil Ecology 73: 116-123.

542. Visser M, Moran J, Regan E, Gormally M, Skeffington MS (2007) The Irish agri-environment: How turlough users and non-users view converging EU agendas of Natura 2000 and CAP. Land Use Policy 24: 362-373.

543. von Haaren C, Reich M (2006) The German way to greenways and habitat networks. Landscape and Urban Planning 76: 7-22.

544. Vos CC, Berry P, Opdam P, Baveco H, Nijhof B, et al. (2008) Adapting landscapes to climate change: examples of climate-proof ecosystem networks and priority adaptation zones. Journal of Applied Ecology 45: 1722-1731.

545. Wagner-Lucker I, Lanz E, Forster M, Janauer GA, Reiter K (2013) Knowledge-based framework for delineation and classification of ephemeral plant communities in riverine landscapes to support EC Habitat Directive assessment. Ecological Informatics 14: 44-47.

546. Walentowski H, Schulze ED, Teodosiu M, Bouriaud O, von Hessberg A, et al. (2013) Sustainable forest management of Natura 2000 sites: a case study from a private forest in the Romanian Southern Carpathians. Annals of Forest Research 56: 217-245.

547. Wamelink GWW, de Knegt B, Pouwels R, Schuiling C, Wegman RMA, et al. (2013) Considerable environmental bottlenecks for species listed in the Habitats and Birds Directives in the Netherlands. Biological Conservation 165: 43-53.

548. Warren MS, Bourn NAD (2011) Ten challenges for 2010 and beyond to conserve Lepidoptera in Europe. Journal of Insect Conservation 15: 321-326.

549. Watzold F, Lienhoop N, Drechsler M, Settele J (2008) Estimating optimal conservation in the context of agri-environmental schemes. Ecological Economics 68: 295-305.

550. Watzold F, Mewes M, van Apeldoorn R, Varjopuro R, Chmielewski TJ, et al. (2010) Cost-effectiveness of managing Natura 2000 sites: an exploratory study for Finland, Germany, the Netherlands and Poland. Biodiversity and Conservation 19: 2053-2069.

551. Wätzold F, Schwerdtner K (2005) Why be wasteful when preserving a valuable resource? A review article on the cost-effectiveness of European biodiversity conservation policy. Biological Conservation 123: 327-338.

552. Weber N, Christophersen T (2002) The influence of non-governmental organisations on the creation of Natura 2000 during the European Policy process. Forest Policy and Economics 4: 1-12.

553. Weingarth K, Heibl C, Knauer F, Zimmermann F, Bufka L, et al. (2012) First estimation of Eurasian lynx (Lynx lynx) abundance and density using digital cameras and capture-recapture techniques in a German national park. Animal Biodiversity and Conservation 35: 197-207.

554. Weiss N, Zucchi H, Hochkirch A (2013) The effects of grassland management and aspect on Orthoptera diversity and abundance: site conditions are as important as management. Biodiversity and Conservation 22: 2167-2178.

555. Welch-Devine M (2012) Searching for Success: Defining Success in Co-Management. Human Organization 71: 358-370.

556. Weslawski JM, Kryla-Straszewska L, Piwowarczyk J, Urbanski J, Warzocha J, et al. (2013) Habitat modelling limitations - Puck Bay, Baltic Sea - a case study. Oceanologia 55: 167-183.

557. Wilson LJ, McSorley CA, Gray CM, Dean BJ, Dunn TE, et al. (2009) Radio-telemetry as a tool to define protected areas for seabirds in the marine environment. Biological Conservation 142: 1808-1817.

558. Winfield IJ, Fletcher JM, James JB, Bean CW (2009) Assessment of fish populations in still waters using hydroacoustics and survey gill netting: Experiences with Arctic charr (Salvelinus alpinus) in the UK. Fisheries Research 96: 30-38.

559. Wingfield R, Murphy K, Gaywood M (2005) Lake habitat suitability for the rare European macrophyte Najas flexilis (Willd.) Rostk. & Schmidt. Aquatic Conservation-Marine and Freshwater Ecosystems 15: 227-241.

560. Wingfield R, Murphy KJ, Gaywood M (2006) Assessing and predicting the success of Najas flexilis (Willd.) Rostk. & Schmidt, a rare European aquatic macrophyte, in relation to lake environmental conditions. Hydrobiologia 570: 79-86.

561. Wjacek J (2008) Benefits and costs of semi-colonial breeding in the Montagu's Harrier Circus pygargus. Belgian Journal of Zoology 138: 36-40.

562. Wood B (2000) Room for nature? Conservation management of the Isle of Rum, UK and prospects for large protected areas in Europe. Biological Conservation 94: 93-105.

563. Wustemann H, Meyerhoff J, Ruhs M, Schafer A, Hartje V (2014) Financial costs and benefits of a program of measures to implement a National Strategy on Biological Diversity in Germany. Land Use Policy 36: 307-318.

564. Young JC, Jordan A, R. Searle K, Butler A, S. Chapman D, et al. (2013) Does stakeholder involvement really benefit biodiversity conservation? Biological Conservation 158: 359-370.

565. Young JC, Jordan A, Searle KR, Butler A, Simmons P, et al. (2013) Framing scale in participatory biodiversity management may contribute to more sustainable solutions. Conservation Letters 6: 333-340.

566. Zacharias I, Parasidoy A, Bergmeier E, Kehayias G, Dimitriou E, et al. (2008) A "DPSIR" model for Mediterranean temporary ponds: European, national and local scale comparisons. Annales De Limnologie-International Journal of Limnology 44: 253-266.

567. Zacharias I, Zamparas M (2010) Mediterranean temporary ponds. A disappearing ecosystem. Biodiversity and Conservation 19: 3827-3834.

568. Zahariev D (2011) Plants With Protection Statute, Endemites And Relicts Of Preslavska Mountain Protected Area. Comptes Rendus De L Academie Bulgare Des Sciences 64: 1285-1290.

569. Zanon B, Geneletti D (2011) Integrating ecological, scenic and local identity values in the management plan of an Alpine Natural Park. Journal of Environmental Planning and Management 54: 833-850.

570. Zografou K, Sfenthourakis S, Pullin A, Kati V (2009) On the surrogate value of red-listed butterflies for butterflies and grasshoppers: a case study in Grammos site of Natura 2000, Greece. Journal of Insect Conservation 13: 505-514.

571. Zohmann M, Pennerstorfer J, Nopp-Mayr U (2013) Modelling habitat suitability for alpine rock ptarmigan (Lagopus muta helvetica) combining object-based classification of IKONOS imagery and Habitat Suitability Index modelling. Ecological Modelling 254: 22-32.

572. Zoppi C (2007) A multicriteria-contingent valuation analysis concerning a coastal area of Sardinia, Italy. Land Use Policy 24: 322-337.
